# A novel class of small-molecule inhibitors targeting bacteriophage infection

**DOI:** 10.1039/d5cb00120j

**Published:** 2025-10-27

**Authors:** Konstantin Plöchl, Thomas Böttcher

**Affiliations:** a Faculty of Chemistry, Institute of Biological Chemistry & Centre for Microbiology and Environmental Systems Science, University of Vienna 1090 Vienna Austria thomas.boettcher@univie.ac.at; b Vienna Doctoral School in Chemistry, University of Vienna 1090 Vienna Austria

## Abstract

Bacteriophages have emerged as important factors in human health and disease, with elevated phage levels associated with exacerbated inflammatory bowel disease, type 2 diabetes and poor outcomes in skin and lung infections. The mechanisms linking phages to these pathologies remain largely unknown, partly because specific chemical tools inhibiting bacteriophage replication (phage blockers) are lacking. Here, we identify benzimidazylpyrazoles as novel bacteriophage antivirals. Unlike existing synthetic antiphage compounds benzimidazylpyrazoles do not intercalate DNA and target an early stage of phage infection after adsorption. An optimized derivative reduced phage titer up to 10^5^-fold and demonstrated activity against different phage morphotypes and bacterial hosts, establishing it as a valuable chemical tool for the study of disease-related phage–host interactions.

## Abstract 2

Discovery of benzimidazylpyrazoles as a new class of synthetic bacteriophage antivirals provides a chemical tool enabling the study of disease-related phage–host interactions.

## Graphical abstract

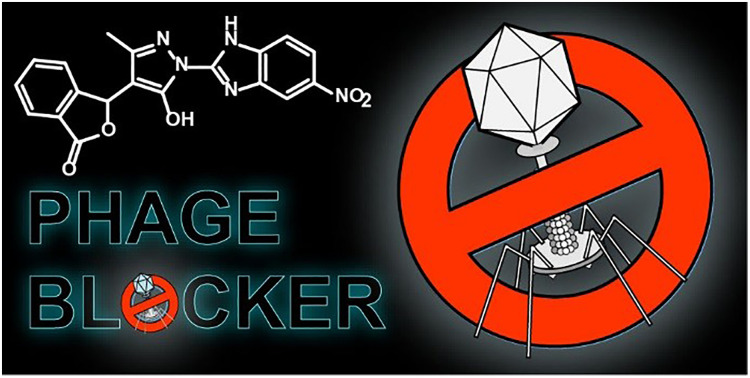

## Introduction

**Fig. 1:**
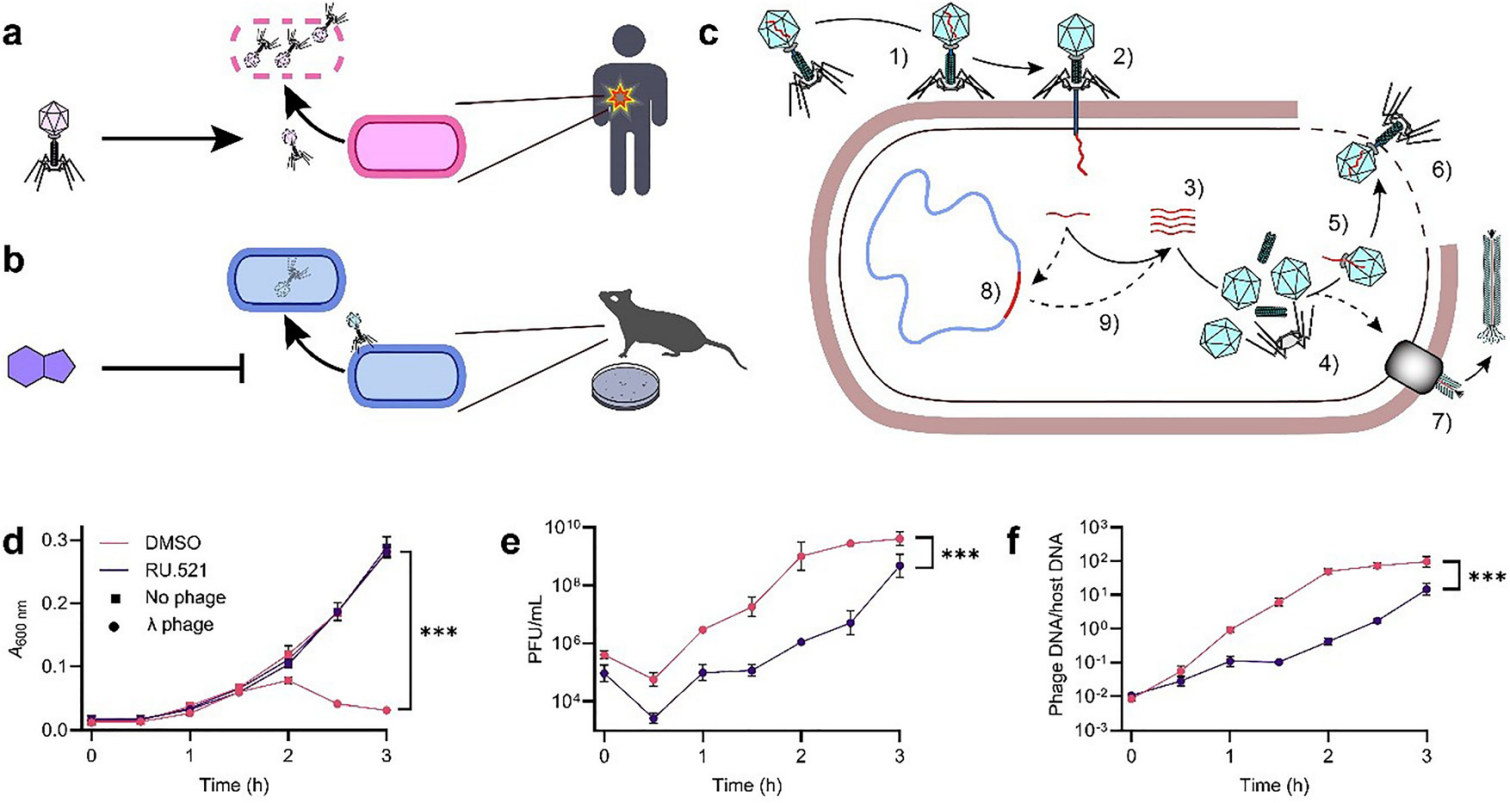
Two alternative paradigms of human phageome modulation. (a) Phage therapy involves the administration of beneficial bacteriophages to selectively lyse pathogenic bacteria infecting the patient. (b) Chemical tools to study how bacteriophages are linked to pathologies are lacking. (c) The bacteriophage infection cycle begins with the adsorption of a virion onto a receptor on the host cell surface (1), followed by injection of the phage genome into the cell (2). Both host- and phage-encoded proteins then mediate viral genome replication (3), capsid synthesis (4) and virion assembly (5). In lytic viruses, mature virions are eventually released through host cell lysis (6). By contrast, filamentous bacteriophages establish a chronic infection, extruding virions continuously without lysing the host (7). Temperate phages can integrate their genome into the host chromosome (8), entering a latent lysogenic state which can revert to the lytic cycle upon prophage induction (9). (d)–(f) The effect of 50 μM RU.521 on *E. coli* DSM 6574 growth (d), phage titer (e) and intracellular viral DNA levels during infection with 0.05 MOI bacteriophage λ in LB with 5 mM MgSO_4_. Data represent mean ± SD of *n* = 3 replicates. Asterisks denote ANOVA time × compound interaction *P*-values of infected samples. MOI, multiplicity of infection; ***P < 0.001.

The human microbiome is a key player in health and disease.^1^ While microbiome research has traditionally focused on bacteria and their relation to host health, the role of bacteriophages – viruses that infect bacteria – has only recently gained attention. This growing interest in the bacteriophage component of the microbiome, known as the phageome, is largely driven by the potential of phage therapy as an alternative to antibiotics. Phage therapy involves the administration of bacteriophages to selectively target and lyse pathogenic bacteria in patients with bacterial infections (Fig. 1a). Although still in the early stages of clinical application, phage therapy has already demonstrated the potential of modulating the human phageome for therapeutic benefit.^2–4^ In contrast, the pathological implications of an overabundance of certain bacteriophages remain underexplored.^5–7^ For instance, patients with inflammatory bowel disease exhibit increased levels of *Caudoviricetes* phages in their gut microbiome,^8–10^ accompanied by distinct alterations in phageome composition specific to either Crohn's disease or ulcerative colitis.^11^ This bacteriophage bloom has been linked to aggravated intestinal inflammation, with bacteriophages isolated from ulcerative colitis patients inducing more interferon γ than those from healthy controls.^12^ Similarly, patients with type 2 diabetes show elevated gut phage titers,^13^ and their bacteriophages elicit a stronger inflammatory response *in vitro.*^14^ In cystic fibrosis patients with *Pseudomonas aeruginosa* lung infections, the presence of Pf bacteriophages is associated with higher bacterial burden, antibiotic resistance and decline in lung function.^15,16^ Additionally, Pf phages promote the persistence of *P. aeruginosa* skin infections and delay wound healing.^17,18^

Despite a growing appreciation for the role of bacteriophages in health, the molecular mechanisms linking phages to poorer disease outcomes remain largely unresolved. A barrier to these investigations has been the lack of chemical tools targeting phage replication (Fig. 1b). Analogous to established antivirals targeting human viruses, an ideal bacteriophage antiviral must inhibit an essential step in the viral infection cycle without affecting the viability of its bacterial host (Fig. 1c). However, existing compounds fall short of this standard. The two principal classes of naturally derived bacteriophage inhibitors, anthracyclines and aminoglycosides, exhibit broad antibacterial activity that precludes their use as phage-specific probes. This activity spectrum is consistent with their proposed evolutionary role as defenses against both bacteriophages and bacterial competitors.^19–21^ Anthracyclines, such as daunorubicin, inhibit phage replication by intercalating viral DNA,^21^ while aminoglycosides, such as kanamycin, interfere with an unknown early step in the infection cycle.^20^ Recent efforts have therefore focused on the discovery of phage-specific antiphage compounds, but all synthetic bacteriophage antivirals discovered thus far are DNA intercalators.^19,21,22^ Here, we report the discovery, chemical optimization and microbiological characterization of a novel class of synthetic bacteriophage antivirals that do not intercalate DNA. An optimized compound from this class demonstrates potent activity against a broad spectrum of phage morphotypes at concentrations well-tolerated by the bacterial host, thereby providing a phage blocker as a chemical tool for studying phage–host interactions of disease-associated bacteriophages.

## Results

### RU.521 inhibits bacteriophage replication

During an unrelated study, we serendipitously observed that RU.521 (1) protected *Escherichia coli* from lysis by bacteriophage λ. Typically, λ infection is characterized by a latent period of host growth, followed by a rapid decline in culture turbidity as host cells lyse (Fig. 1d). However, the addition of 50 μM RU.521 prevented this decline, allowing host growth to continue unperturbed for three hours (Fig. 1d). Motivated by this unexpected finding, we monitored phage titers in culture supernatants during the infection process (Fig. 1e). RU.521 consistently suppressed phage titers across all measured time points between 4- and 900-fold. Titers initially decreased in both RU.521-treated and control samples due to phage adsorption onto host cells. Subsequent phage propagation led to a rise in titer above initial levels within one hour in untreated samples, whereas this increase was delayed until two hours in treated samples (Fig. 1e). To characterize the effect on phage genome replication, we tracked the ratio of intracellular phage DNA to host DNA throughout the course of infection using qPCR (Fig. 1f). Again, RU.521 consistently decreased phage DNA levels within host cells up to 120-fold. Collectively, RU.521 demonstrated inhibition of λ across three hallmarks of phage infection, establishing it as a genuine bacteriophage antiviral.

### Chemical modification of RU.521 yields a broad-spectrum antiviral

**Fig. 2:**
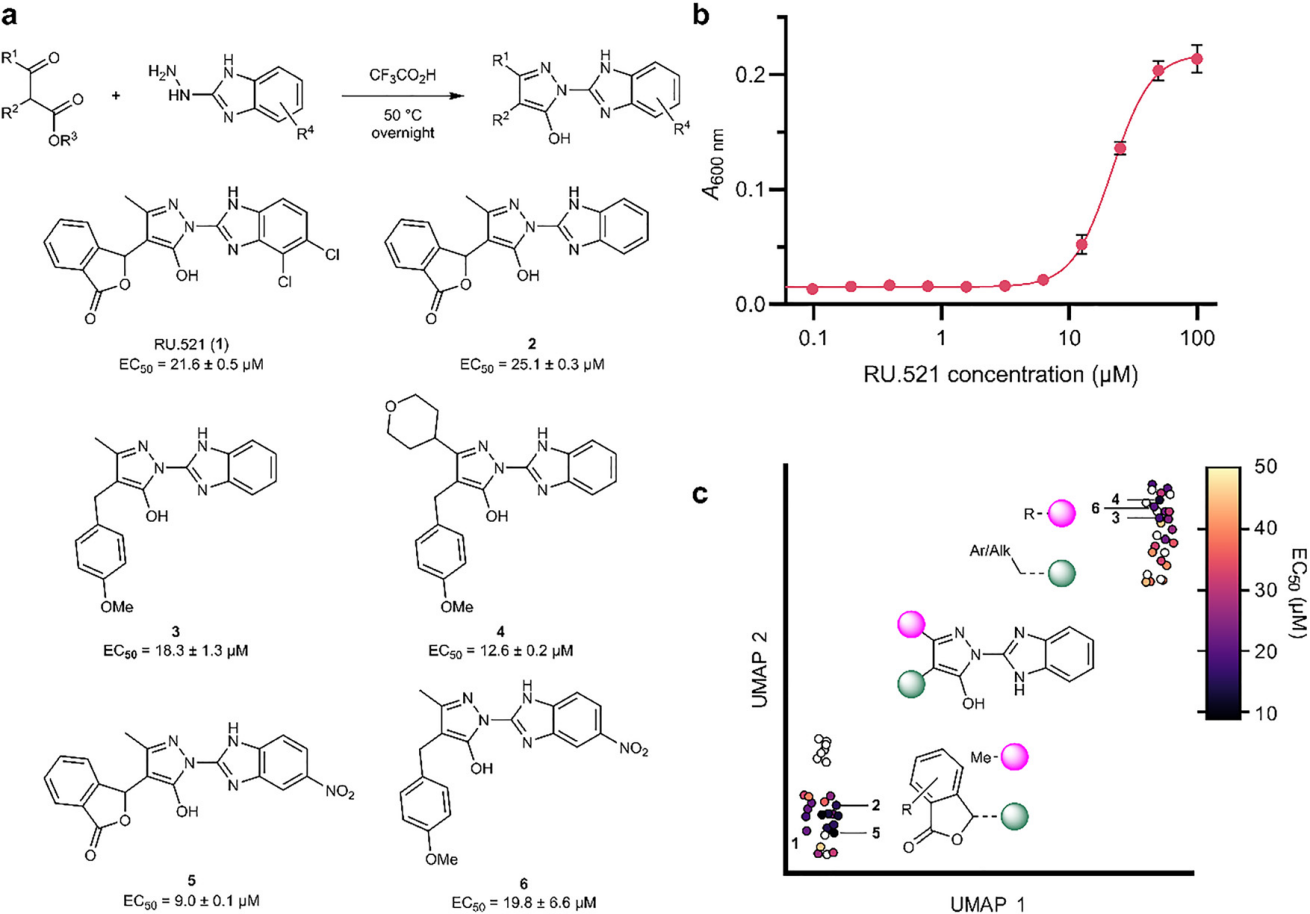
Structure–activity relationship of benzimidazylpyrazole phage blockers. (a) General synthesis and structures of benzimidazylpyrazole bacteriophage antivirals. (b) *A*_600 nm_ of *E. coli* DSM 6574 cultures in LB with 5 mM MgSO_4_ infected with 0.2 MOI bacteriophage λ after 3 h incubation in presence of RU.521. (c) UMAP embedding of molecular fingerprints of the 56 tested compounds, color-coded by EC_50_, shows two distinct clusters of active phage blockers. Empty circles denote EC_50_ > 50 μM. Data in (b) represent mean ± SD of *n* = 3 replicates with 4-parameter logistic regression. EC_50_ values in (a) are ± SE calculated from *n* = 3 replicates. MOI, multiplicity of infection.

We next probed the structure–activity relationship of RU.521 to develop a more potent phage antiviral. We used a turbidimetric assay based on the protection against host lysis to measure dose–response relationships (Fig. 2b). The benzimidazylpyrazole scaffold of RU.521 comprises three linked heterocycles (Fig. 2a), each of which we modified individually. We tested 56 compounds based on the deschloro analog 2 because of better synthetic access and commercial availability (Fig. 2c and Table S3). Exchanging the western isobenzofuranone with various aryl and aliphatic moieties retained antiviral activity, with 4-methoxybenzyl (3) yielding the most potent derivative in this series (Fig. 2a). Substitutions at C5 (4), but not C3, of the central pyrazole further reduced EC_50_. The eastern benzimidazole was necessary for activity since removal of the imidazole H-bond donor was not tolerated. Addition of substituents to the benzene ring of the benzimidazole created another active series and furnished 5, with single-digit micromolar potency (Fig. 2a). However, combining the best western and eastern fragments (6) did not further increase potency. Active antiphage compounds were well tolerated by the bacterial host (Table S3); and host toxicity did not correlate with antiviral activity (Fig. S1).

**Fig. 3:**
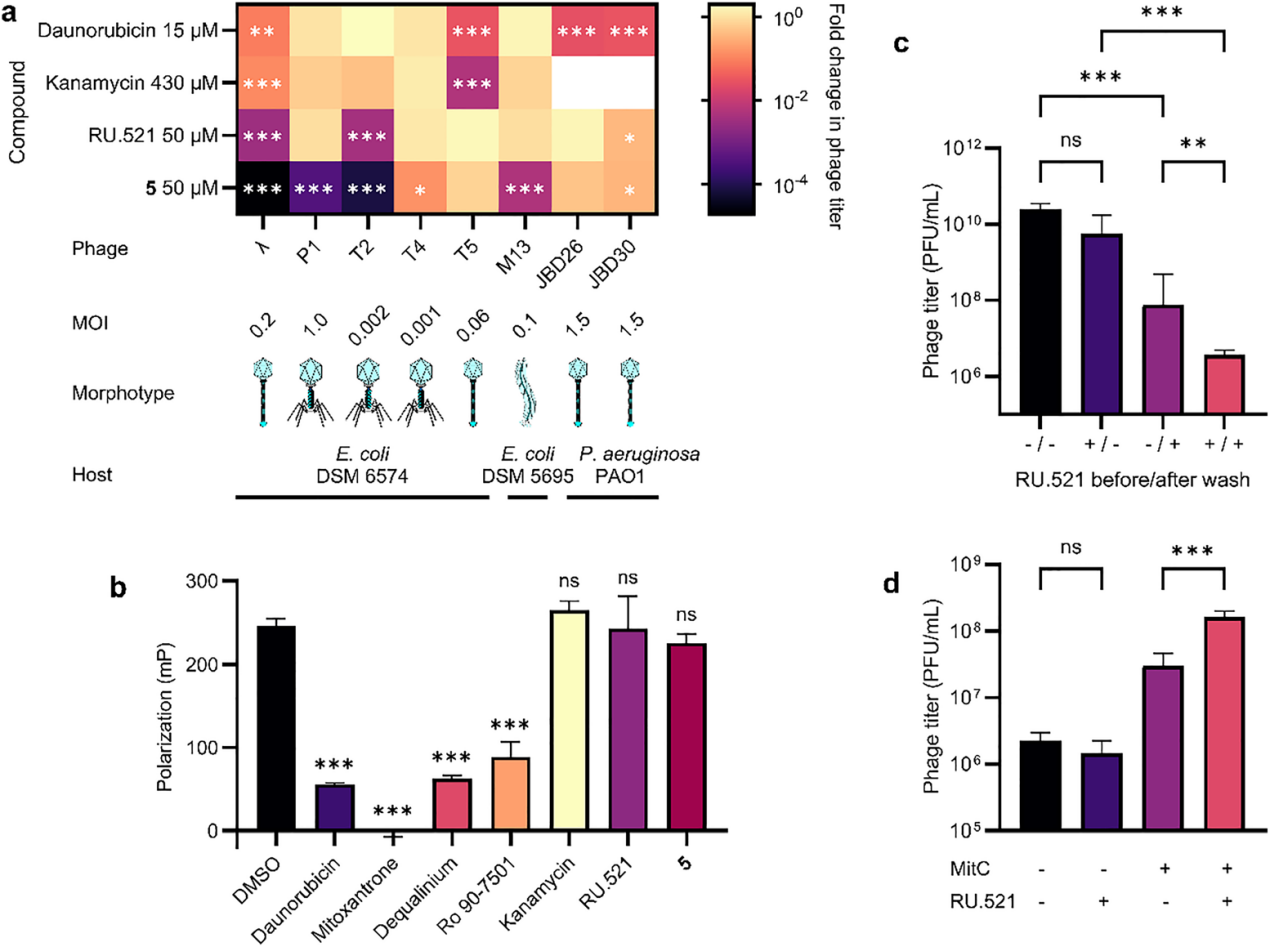
An optimized phage antiviral acts across diverse phage morphotypes at an early stage of infection. (a) Activity spectra for six *E. coli* and two *P. aeruginosa* phages representing siphovirus (λ, T5, JBD26, JBD30), myovirus (P1, T2, T4) and inovirus (M13) morphotypes. Colors indicate fold change in phage titer *versus* DMSO after 2 h incubation. (b) DNA intercalation assay with 30 μM bacteriophage antivirals. (c) Effect on phage titer of 50 μM RU.521 before or after incubation of *E. coli* DSM 6574 with 0.05 MOI bacteriophage λ. (d) Effect of 50 μM RU.521 on phage titer following induction of the λ lysogen *E. coli* DSM 8589 using 1.5 μM mitomycin C. Colors in (a) represent means of *n* = 3 replicates. ANOVA with Dunnett's test (Šidák's test for kanamycin). Data in (b) show mean ± SD of *n* = 5 replicates. ANOVA with Dunnett's test. Data in (c) and (d) represent mean ± SD of *n* = 3 replicates. ANOVA with Fisher's LSD test (c) or Šidák's test (d). MitC, mitomycin C; MOI, multiplicity of infection; ns, not significant; *P < 0.05; **P < 0.01; ***P < 0.001.

To test whether RU.521 and its optimized derivative 5 exhibit antiviral activity against other phages than λ, we determined the reduction in phage titer on a panel of six *E. coli* viruses and two *P. aeruginosa* viruses representing siphovirus, myovirus and inovirus morphotypes (Fig. 3a). For comparison, we included daunorubicin and kanamycin, two established bacteriophage antivirals, at their reported concentrations (15 μM for daunorubicin,^21^ 430 μM for kanamycin^20^), and tested RU.521 and 5 at 50 μM. In cases requiring kanamycin resistance, we transformed the *E. coli* host with a plasmid encoding aminoglycoside phosphotransferase. Consistent with prior studies,^20,21^ daunorubicin inhibited the replication of λ, T5, JBD26 and JBD30 but not M13, while kanamycin was active only against λ and T5 (Fig. 3a). (Kanamycin was not tested on *P. aeruginosa* phages due to its bactericidal effect on the host.) In contrast, RU.521 inhibited λ, T2, and *P. aeruginosa* phage JBD30, while its derivative 5 was additionally active against phages P1, T4 and M13. The modest potency increase of 5 in the lysis protection assay translated into an up to 10^5^-fold reduction in viral titer. Compound 5 showed activity across all tested morphotypes and is the first^19^ antiviral against the single-stranded DNA (ssDNA) virus M13. Only one other inhibitor of any inovirus has been reported so far,^19^ but this finding^23^ from 1964 could not be replicated in a study^20^ from 2022.

### RU.521 inhibits an early step of phage infection after adsorption

We hypothesized that 5's broader activity spectrum compared to RU.521 resulted from its enhanced potency rather than a different mechanism of action. Given the unique inhibition of M13, with its ssDNA genome, we suspected that benzimidazylpyrazole phage blockers act through a mechanism distinct from that of anthracyclines, which intercalate double-stranded DNA.^21^ To test this hypothesis, we measured DNA intercalation *via* the displacement of a DNA-binding dye. As expected, daunorubicin and the related anthraquinone mitoxantrone demonstrated potent intercalation (Fig. 3b). Similarly, the hitherto uncharacterized synthetic bacteriophage antivirals^21^ dequalinium chloride (a disruptor of bacterial membranes^24^) and Ro 90-7501 (originally developed as a protein phosphatase 5 inhibitor^25^) intercalated DNA as well (see Fig. S2 for chemical structures). In contrast, neither kanamycin, RU.521 nor 5 exhibited intercalation, suggesting that they act *via* distinct mechanisms.

We then sought to identify the step of the infection cycle targeted by benzimidazylpyrazoles. Because 5 inhibited the replication of both exclusively lytic (*e.g.*, T2) and chronic (M13) phages (Fig. 3a), host lysis is not the targeted step. We therefore started by examining the effect of RU.521 on phage adsorption. Host cells were incubated statically with λ and RU.521 for 15 minutes to allow phage adsorption. After washing to remove unbound virions and RU.521, the cells were incubated with a fresh dose of RU.521 or vehicle for three hours, and phage titers were measured. Notably, RU.521 addition after, but not before, washing was sufficient to significantly suppress phage titer (Fig. 3c), with 83% of titer variation stemming from RU.521 presence after washing (ANOVA *P* < 0.001), indicating that it acts after virion adsorption. Lastly, we examined RU.521's effect on prophage induction. Induction of a λ lysogen using mitomycin C^26^ led to a rise in phage titer that was not prevented by RU.521 (Fig. 3d), suggesting that it acts before viral genome replication and transcription of lytic genes. Together, these results demonstrate that RU.521 acts at an early step of the infection cycle immediately after phage adsorption.

## Discussion

Bacteriophages shape bacterial community composition across various natural ecosystems.^27^ The strong selective pressure phages exert on their bacterial hosts has fueled an evolutionary arms race, resulting in the development of diverse bacterial defense strategies, including the production of metabolites that inhibit phage infection.^19,28^ The two major classes of secreted bacteriophage antivirals – anthracyclines and aminoglycosides – not only provide defense against phage infection but also function as antibacterials that inhibit the growth of bacterial competitors. In order to develop a phage-specific chemical tool, we have presented the first class of synthetic antiphage compounds that do not intercalate DNA. This distinct feature of benzimidazylpyrazoles probably explains their activity against ssDNA phages, which are unaffected by anthracyclines and all other synthetic bacteriophage antivirals. Given that RU.521 acts after adsorption, it is likely taken up by host cells, indicating an intracellular mode of action. The inhibition of diverse phages suggests that the target is probably not phage-specific but rather host-encoded and conserved across *E. coli* and *P. aeruginosa.*

RU.521 was originally developed as an inhibitor of murine cyclic GMP-AMP synthase (cGAS) and could therefore potentially interfere with the synthesis of related signaling molecules in bacteria. cGAS is part of the cGAS/STING antiviral immune pathway in animals,^29^ which shares an evolutionary ancestry with the cyclic-oligonucleotide-based antiphage signalling system (CBASS) found in many bacteria.^30^ However, since the *E. coli* strain DSM 6574 used in our study lacks CBASS,^31^ interference with it can be excluded. Subsequent optimization of RU.521 yielded a potent derivative active against all three tested phage morphotypes. The compound demonstrated selectivity for active lytic or chronic phage infections without affecting the induction of lysogenic λ prophage. This broad activity with regard to phage morphotype and bacterial host but selectivity with regard to infection state makes it a valuable tool for studying phage–host interactions.

## Methods

### Chemistry

Details on the chemical compounds used, including their commercial sources, synthesis and characterization, are provided in the SI.

### Bacterial strains and phages

*E. coli* strains DSM 6574, DSM 5695 and DSM 8589 and *P. aeruginosa* PAO1 (DSM 22644) were cultured in LB medium (Carl Roth X964.4) at 37 °C with shaking at 180 rpm unless otherwise noted. *E. coli* DSM 6574 and DSM 5695 were transformed with plasmid pDSP1 (Addgene 199384) to provide kanamycin resistance when needed. All experiments were performed using cultures diluted after overnight incubation. All phages used, their sources and hosts are listed in Table S1. Bacteriophage λ was isolated from the lysogen *E. coli* DSM 8589 by induction with 1.5 μM mitomycin C and filter-sterilizing (0.2 μm) the culture supernatant after 4 h of incubation. Phage lysates were prepared by propagating the phage with its host until lysis was observed (or overnight for M13). After adding CHCl_3_, the cultures were incubated for an additional 10 min, then centrifuged at 3000 × *g* and 4 °C for 10 min. The aqueous supernatants were filter-sterilized twice and stored above CHCl_3_ at 4 °C. Phage titers were determined by spot assay,^32^ where 3 μL of serial 10-fold dilutions in SM buffer (50 mM Tris/HCl pH 7.5, 100 mM NaCl, 8 mM MgSO_4_) were spotted on host bacterial lawns in top agar (LB + 6 g L^−1^ agar) overlaid on bottom agar (LB + 10 mM CaCl_2_ + 15 g L^−1^ agar). Plates were incubated overnight at 37 °C, and plaques were counted the following day.

### Phage λ infection monitoring

Cultures of *E. coli* DSM 6574 (OD = 0.1), bacteriophage λ (MOI = 0.05) and RU.521 (50 μM) or DMSO (0.5 vol%) in 15 mL LB + 5 mM MgSO_4_ were incubated in 50-mL tubes for 3 h. 1-mL samples were collected every 30 min in 1.5-mL tubes, 100 μL of which was transferred to a flat transparent 96-well plate (Sarstedt 82.1581.001) for absorbance measurements at 600 nm (Tecan Infinite 200 Pro). The remaining samples were centrifuged at 7000 × *g* and 4 °C for 5 min. The supernatants were filter-sterilized and stored at 4 °C for subsequent phage titer determination. To isolate intracellular DNA for qPCR, the cell pellets were resuspended in 1 mL PBS (Carl Roth 0890.1) and centrifuged at 7000 × *g* and 4 °C for 5 min. The supernatants were discarded, and the cell pellets were resuspended and centrifuged again to remove residual virions. After discarding the supernatants, the twice washed cell pellets were stored at −20 °C overnight. On the next day, DNA was extracted using DNeasy Blood & Tissue Kit (Qiagen 69504), concentrated in vacuum at 45 °C for 2 h (Eppendorf Concentrator Plus) and dissolved in 20 μL H_2_O. DNA concentrations were measured photometrically (Tecan Spark with NanoQuant plate) and adjusted to 1 ng μL^−1^ with H_2_O. qPCR was performed using 250 pg μL^−1^ DNA template, 250 nM primers (Table S2) and 20 μL 1 × Luna mix (NEB M3003E) in a white 96-well PCR plate (Brand 781364) on a LightCycler 96 (Roche) with detection at 470 nm/510 nm. Samples were preincubated at 95 °C for 60 s, followed by 40 cycles of amplification (95 °C for 15 s, then 60 °C for 60 s) and high-resolution melting analysis. Abundance of λ tail gene *V* was normalized to host gene *gyrA*.

### Antiphage dose–response assay

Cultures of *E. coli* DSM 6574 (OD = 0.1), bacteriophage *λ* (MOI = 0.2) and 2-fold serial dilutions of antiphage compound or DMSO (1 vol%) in 100 μL LB + 5 mM MgSO_4_ were incubated in flat transparent 96-well plates for 3 h before measuring absorbance at 600 nm. EC_50_ values were calculated using 4-parameter logistic regression (GraphPad Prism 10). Data points where host toxicity led to a decrease in absorbance at high compound concentrations (50, 100 μM) were excluded from analysis.

### Host growth inhibition assay

Cultures of *E. coli* DSM 6574 (OD = 0.1) and antiphage compound (50 μM) or DMSO (1 vol%) in 100 μL LB + 5 mM MgSO_4_ were incubated in flat transparent 96-well plates for 3 h before measuring absorbance at 600 nm, and the relative differences in absorbance to DMSO control were calculated. The EC_50_ values of active antiphage compounds (EC_50_ < 50 μM) were correlated to host growth inhibition using linear regression.

### UMAP embedding of benzimidazylpyrazole phage blockers

Chemical structures were loaded into Maestro 13.4 (Schrödinger), and tautomers were generated at pH 7.4 using Epik. Only the *R*-enantiomers of isobenzofuranones were considered. The most prominent tautomer for each structure was exported and used to generate 1024-bit Morgan fingerprints using the package rdkit in Python 3. The UMAP embedding was calculated from the fingerprints using the package umap.

### Antiphage activity spectra

Cultures of host bacteria (OD = 0.1), phage (see Fig. 3a for MOI) and compound (15 μM daunorubicin hydrochloride (TCI D4532), 430 μM kanamycin sulfate (Carl Roth T832.2), 50 μM RU.521, 50 μM 5) or DMSO (0.5 vol%) in 100 μL LB + 5 mM MgSO_4_ were incubated in flat transparent 96-well plates for 2 h. (No Mg^2+^ was added for kanamycin and its control because it interferes with aminoglycoside antiphage activity.^33,34^) After centrifugation at 7000 × *g* and 4 °C for 10 min, the supernatants were filter-sterilized, and their phage titers were determined.

### DNA intercalation assay

The method was adapted from a reported procedure.^35^ Antiphage compounds (30 μM) or DMSO (0.3 vol%), acridine orange (50 nM, Carl Roth 7632.1) and salmon sperm DNA (6 mg L^−1^, Carl Roth 5434.1) were incubated in 100 μL HEN buffer (10 mM HEPES pH 7.5, 1 mM EDTA, 100 mM NaCl) in a flat black 96-well plate (Thermo Scientific 611F96BK) at 25 °C with shaking at 180 rpm in the dark for 30 min. Then, fluorescence polarization was recorded at 485 nm/535 nm (Tecan Spark).

### Phage adsorption assay

Cultures of *E. coli* DSM 6574 (OD = 0.1), bacteriophage λ (MOI = 0.05) and RU.521 (50 μM) or DMSO (0.5 vol%) in 1 mL LB + 5 mM MgSO_4_ were incubated statically at ambient temperature in 1.5-mL tubes for 15 min. Then, the cultures were centrifuged at 7000 × *g* and 25 °C for 3 min. The supernatants were discarded, and the cell pellets were resuspended in 1 mL LB + 5 mM MgSO_4_, followed by another round of centrifugation, decantation and resuspension. 100 μL of washed cultures were transferred to a 96-well flat transparent plate, and RU.521 (50 μM) or DMSO (0.5 vol%) were added. After incubation at 37 °C with shaking at 180 rpm for 3 h, the samples were centrifuged at 7000 × *g* and 4 °C for 10 min. The supernatants were filter-sterilized, and their phage titers were determined.

### Lysogen induction assay

Cultures of *E. coli* DSM 8589 (OD = 0.1), mitomycin C (1.5 μM, Roche 10107409001) and/or RU.521 (50 μM) or DMSO (0.5 vol%) in 100 μL LB + 5 mM MgSO_4_ were incubated in a flat transparent 96-well plate for 4 h. After centrifugation at 7000 × *g* and 4 °C for 10 min, the supernatants were filter-sterilized, and their phage titers were determined.

## Conflicts of interest

There are no conflicts to declare.

## Supplementary Material

CB-OLF-D5CB00120J-s001

## Data Availability

The data supporting this article have been included as part of the supplementary information (SI). Supplementary information is available. See DOI: https://doi.org/10.1039/d5cb00120j.
