# Supplementary material for: A novel class of small-molecule inhibitors targeting bacteriophage infection
Source: RSC Chem Biol. 2025 Oct 27;7(1):31–7. doi: 10.1039/d5cb00120j (PMC12587450; doi:10.1039/d5cb00120j)
Supplement: CB-007-D5CB00120J-s001 [file CB-007-D5CB00120J-s001.pdf]

Supplementary Information (SI) for RSC Chemical Biology.
This journal is © The Royal Society of Chemistry 2025

Supplementary information

for

A novel class of small-molecule inhibitors targeting bacteriophage infection

Konstantin Plöchl[a,b] & Thomas Böttcher[a,*]

[a]Faculty of Chemistry, Institute of Biological Chemistry & Centre for Microbiology and Environmental Systems Science, University of Vienna, 1090 Vienna, Austria

[b]Vienna Doctoral School in Chemistry, University of Vienna, 1090 Vienna, Austria

*thomas.boettcher@univie.ac.at

Tab. S1 | Bacteriophages used in this study.

Name	Host	Source
λ	*E. coli* DSM 6574	Induced from *E. coli* DSM 8589
P1	*E. coli* DSM 6574	DSM 5757
T2	*E. coli* DSM 6574	DSM 16352
T4	*E. coli* DSM 6574	DSM 4505
T5	*E. coli* DSM 6574	DSM 16353
M13	*E. coli* DSM 5695	DSM 13976
JBD26	*P. aeruginosa* PAO1	Gift from Joseph Bondy-Denomy[1]
JBD30	*P. aeruginosa* PAO1	Gift from Joseph Bondy-Denomy[1]

Tab. S2 | DNA primers for qPCR. All oligonucleotides were ordered from Metabion.

**Name**	**Sequence**
V forward	AAGATCGCAGCACGGTAACA
V reverse	GTTTTATCCGCAGACACCGC
gyrA forward	ACGCGACTTGGTTGGGTATT
gyrA reverse	GTCTCTCTGATCGTGCCTCG

Tab. S3 | Antiphage activity of benzimidazylpyrazoles. Data were calculated from *n* = 3 replicates.

ID	Structure	EC_{50} ± SE (µM)	Host growth inhibition at 50 µM (mean ± SE)	Source
**1** (RU.521)	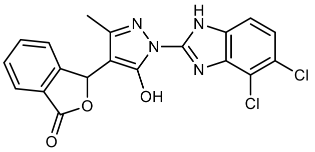	21.6 ± 0.5	21.6% ± 0.8%	BLD BD00773267
**2**	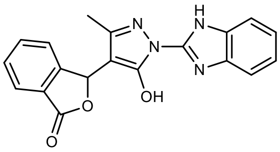	25.1 ± 0.3	16.0% ± 2.5%	ChemDiv Y600-1720
**7**	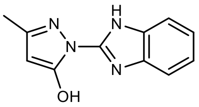	> 50	13.7% ± 1.3%	Ambinter 1064532
**8**	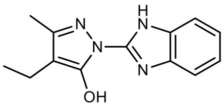	40.0 ± 2.7	27.9% ± 0.5%	Ambinter 1901992
**9**	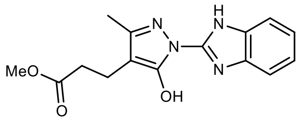	> 50	11.4% ± 0.8%	Ambinter 8242333
**10**	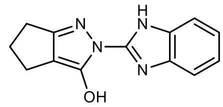	> 50	13.6% ± 0.8%	This work
**11**	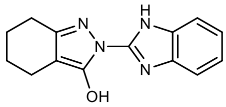	37.7 ± 4.9	22.8% ± 0.6%	Ambinter 1901999
**12**	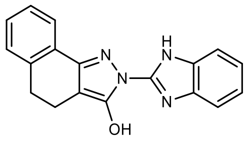	43.8 ± 0.6	3.4% ± 3.0%	This work
**13**	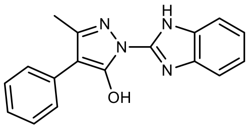	39.9 ± 8.8	19.2% ± 0.7%	Ambinter 38760247
**14**	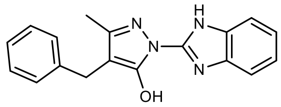	22.2 ± 0.3	21.2% ± 0.7%	This work
**15**	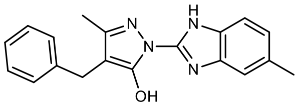	26.4 ± 0.7	20.5% ± 1.0%	Ambinter 30450581
**16**	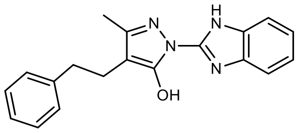	31.9 ± 1.9	9.4% ± 1.6%	Ambinter 2130126

**17**	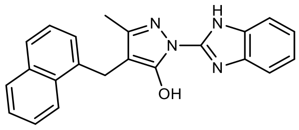	32.2 ± 1.4	15.1% ± 0.9%	Ambinter 1573637
**18**	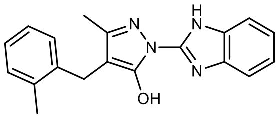	40.4 ± 5.0	23.9% ± 2.3%	Ambinter 1901998
**19**	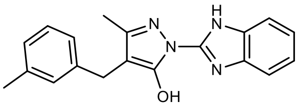	34.9 ± 1.6	21.0% ± 2.0%	Ambinter 1901997
**20**	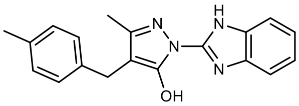	24.6 ± 0.2	18.7% ± 1.6%	Ambinter 1901995
**21**	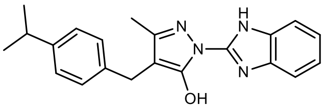	> 50	6.9% ± 1.1%	Ambinter 1573639
**22**	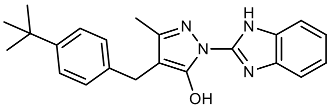	> 50	4.2% ± 1.3%	Ambinter 1573640
**23**	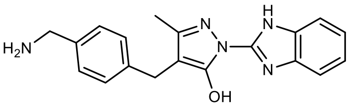	> 50	15.4% ± 0.6%	This work
**24**	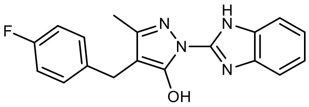	30.4 ± 1.4	13.0% ± 1.3%	This work
**25**	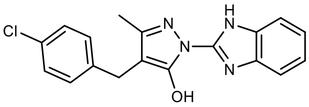	19.9 ± 7.1	22.3% ± 1.7%	Ambinter 1901996
**26**	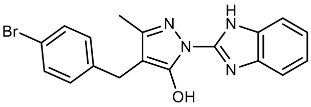	22.7 ± 0.4	4.6% ± 0.8%	Ambinter 1911330
**27**	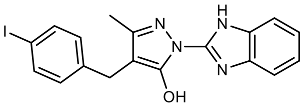	45.2 ± 8.8	16.6% ± 1.1%	This work
**28**	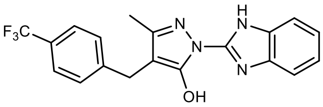	19.0 ± 0.6	6.3% ± 2.6%	This work
**3**	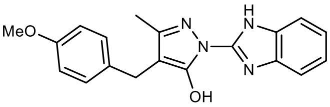	18.3 ± 1.3	13.1% ± 1.8%	This work
**29**	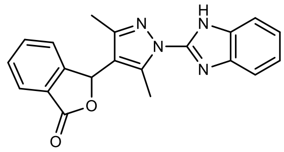	> 50	-2.9% ± 0.6%	This work
**30**	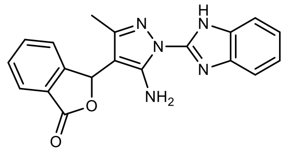	> 50	0.2% ± 1.6%	This work

**31**	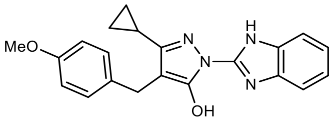	30.2 ± 2.9	4.9% ± 1.6%	This work
**32**	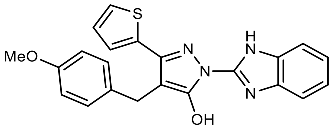	> 50	20.8% ± 1.3%	This work
**4**	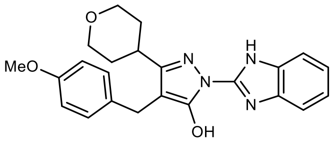	12.6 ± 0.2	25.0% ± 0.8%	This work
**33**	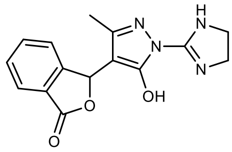	> 50	-2.2% ± 0.7%	This work
**34**	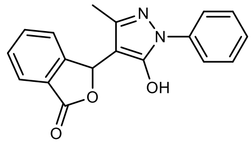	> 50	-2.9% ± 1.4%	Ambinter 19793492
**35**	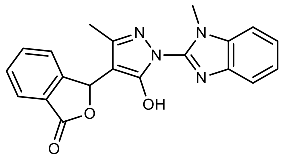	> 50	18.9% ± 0.6%	This work
**36**	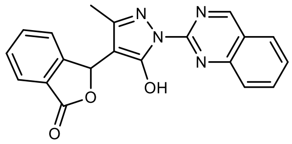	> 50	15.2% ± 0.7%	This work
**37**	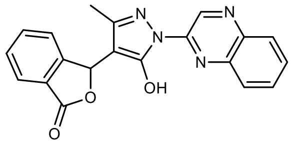	> 50	8.7% ± 0.8%	This work
**38**	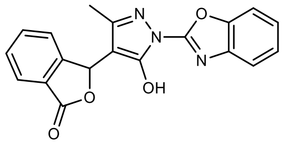	> 50	-2.0% ± 0.6%	This work
**39**	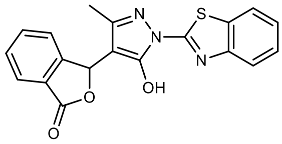	> 50	19.0% ± 0.8%	This work
**40**	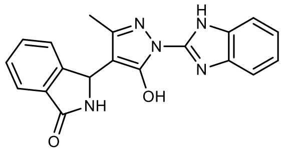	31.0 ± 1.3	14.7% ± 0.8%	This work
**41**	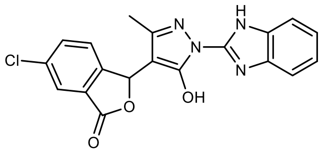	26.8 ± 0.5	18.2% ± 1.1%	This work

**42**	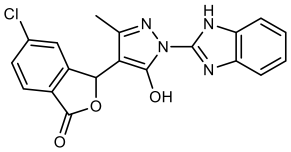	45.8 ± 13.4	17.9% ± 0.8%	This work
**43**	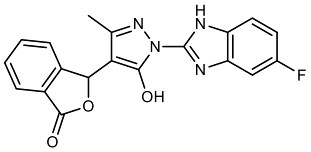	13.8 ± 0.6	11.9% ± 1.4%	This work
**44**	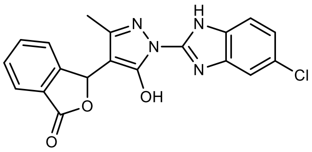	14.7 ± 0.4	13.7% ± 0.7%	This work
**45**	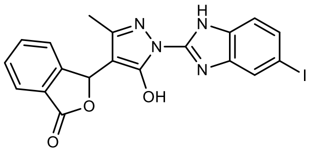	15.7 ± 0.9	22.4% ± 0.9%	This work
**46**	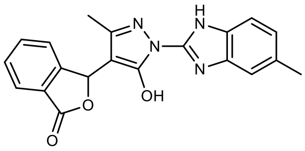	12.9 ± 0.5	15.4% ± 0.9%	This work
**47**	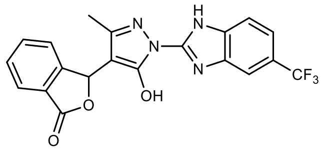	15.5 ± 1.3	20.9% ± 0.4%	This work
**48**	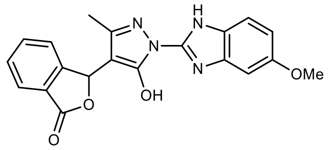	23.8 ± 2.0	8.9% ± 0.8%	This work
**5**	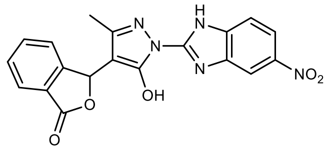	9.0 ± 0.1	14.7% ± 0.9%	This work
**49**	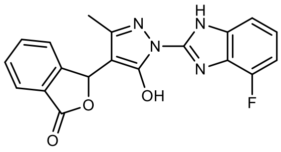	39.3 ± 0.8	18.2% ± 0.7%	This work
**50**	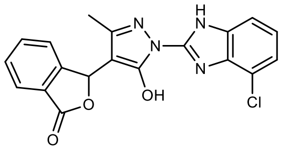	18.1 ± 0.3	12.2% ± 1.4%	This work
**51**	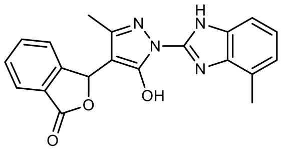	32.8 ± 0.3	9.6% ± 0.9%	This work

**52**	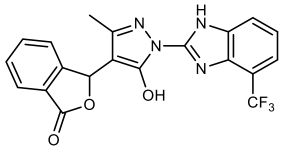	21.6 ± 0.4	14.1% ± 0.8%	This work
**53**	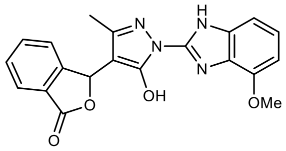	21.5 ± 0.2	7.0% ± 1.2%	This work
**54**	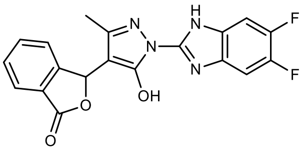	36.2 ± 2.6	22.1% ± 0.6%	This work
**55**	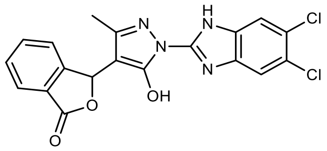	9.5 ± 0.4	26.4% ± 1.3%	This work
**6**	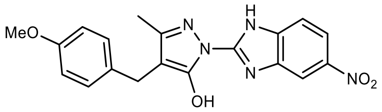	19.8 ± 6.6	11.0% ± 2.9%	This work
**56**	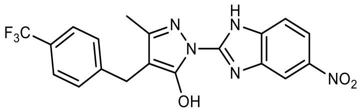	> 50	8.3% ± 3.4%	This work

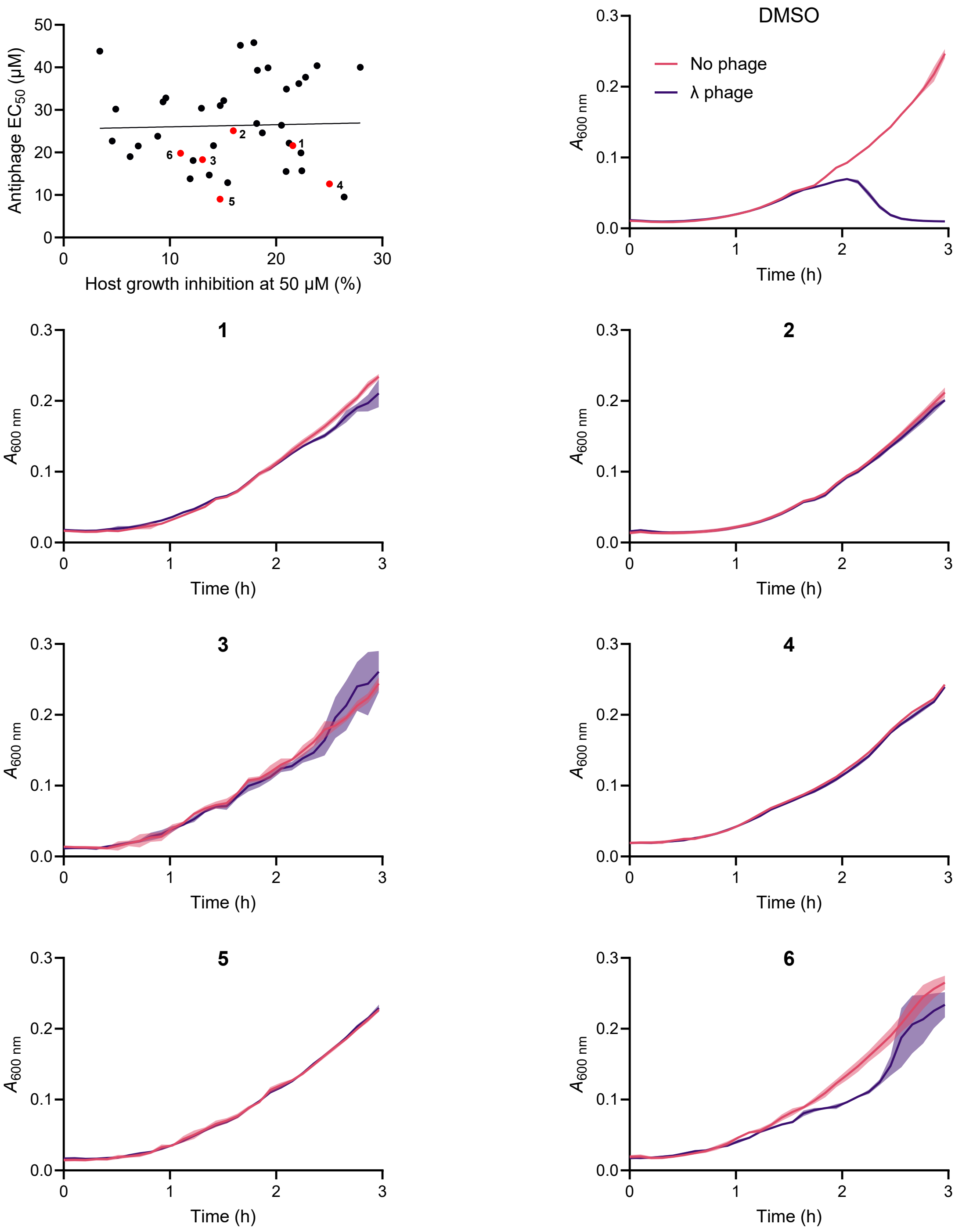

Fig. S1 | Antiphage activity is unrelated to host toxicity. Top left: Correlation between antiphage EC_{50} and inhibition of *E. coli* DSM 6574 growth ($A_{600\ nm}$) relative to DMSO after 3 h incubation with 50 µM compound. Line shows linear regression. Pearson's R^2 = 0.0008. Compounds depicted in Fig. 2a are highlighted in red. Other panels: Growth curves of *E. coli* DSM 6574 in LB with 5 mM $MgSO_4$ with 50 µM of indicated compounds in presence or absence of 0.2 MOI bacteriophage λ. Curves show mean ± SD of ***n*** = 3 replicates. MOI, multiplicity of infection.

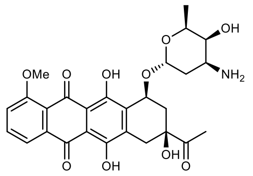

Daunorubicin

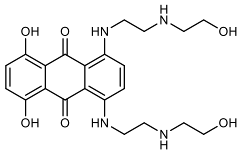

Mitoxantrone

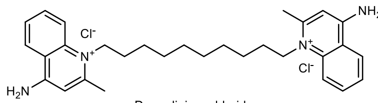

Dequalinium chloride

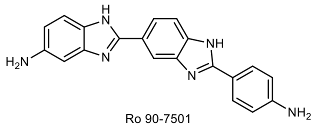

Ro 90-7501

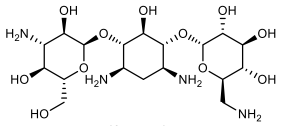

Kanamycin

Fig. S2 | Structures of bacteriophage antivirals tested for DNA intercalation.

Chemical synthesis

All reagents and solvents were obtained from commercial suppliers and used without further purification. Commercial sources of starting materials are detailed when first mentioned. Where indicated, moisture-sensitive reactions were carried out under N_2 in dry solvents using heat-dried glassware on a Schlenk line. Flash chromatography was performed on a CombiFlash NextGen 100 (Teledyne Isco) with RediSep Silver SiO_2 columns. Eluent gradients are denoted as vol% of the second solvent. Preparative HPLC was performed on a SpotPrep (Armen Instruments) using a Reprosil 100 C_{18} column (Dr. Maisch r10.96.s2520, dimensions 250 × 20 mm, particle size 10 µm) with 10 mL/min flow rate. HRMS was measured on a timsTOF flex (Bruker) with electrospray ionization (ESI). Mass-to-charge ratios (*m*/*z*) are reported in Da/*e*. NMR spectra were recorded on AV III 600, AV III 400 and AV III 200 spectrometers (all Bruker) in deuterated solvents at 298 K unless otherwise noted and were processed with MestReNova 14.3. Chemical shifts are reported in ppm and are referenced to residual proton signals of deuterated solvents. Signal multiplicities are abbreviated as: s = singlet, d = doublet, t = triplet, q = quartet, m = multiplet. Intermediate exchange tautomerism of benzimidazoles led to ^{13}C signal broadening.[2] NMR spectra were therefore recorded at elevated temperatures to aid coalescence; still, not all ^{13}C signals could be observed and had to be inferred from HMBC experiments.

General method A (hydrazinolysis)

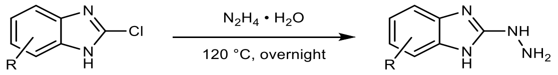

A pressure tube was charged with the 2-chlorobenzimidazole and hydrazine (2 mL, 51 wt% in H_2O). The tube was sealed, and the reaction mixture was stirred at 120 °C overnight. The reaction mixture was allowed to cool, adsorbed onto SiO_2 and concentrated in vacuum. The crude was purified by flash chromatography to afford the 2-hydrazinylbenzimidazole.

General method B (2-benzylacetoacetate synthesis)

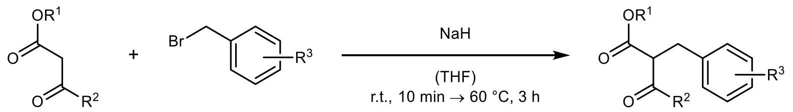

NaH (1.20 equiv, 60 wt% in mineral oil) was suspended in dry THF (500 mM) under N_2. The acetoacetate ester (1.20 equiv.) was added dropwise, and the reaction mixture was stirred for 5 min. The benzyl bromide (1.00 equiv.) was added in one portion, and the reaction mixture was stirred at r.t. for 10 min, then at 60 °C for 3 h. The reaction mixture was allowed to cool, diluted with sat. aq. NH_4Cl (15 mL) and extracted with CH_2Cl_2 (3 × 15 mL). The

combined org. phases were dried over Na_2SO_4 and concentrated in vacuum. The crude was purified by flash chromatography to afford the 2-benzylacetoacetate ester.

General method C (pyrazole synthesis)

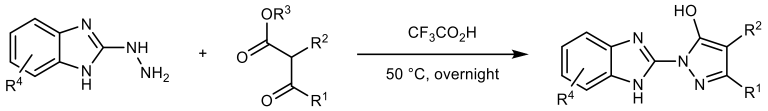

The 2-hydrazinylbenzimidazole and the (substituted) acetoacetate ester were stirred in CF_3CO_2H (500 µL) at 50 °C overnight. The reaction mixture was allowed to cool and concentrated in vacuum. The crude was purified by HPLC to afford the 1-(benzimidazol-2-yl)pyrazol-3-ol. When R^2 is an isobenzofuranone, products were afforded as racemates.

HPLC method 1

H_2O/MeCN 5→95% + 0.1% HCO_2H

HPLC method 2

H_2O/MeOH 20→95% + 0.1% HCO_2H

2-(1*H*-Benzo[*d*]imidazol-2-yl)-2,4,5,6-tetrahydrocyclopenta[*c*]pyrazol-3-ol (10)

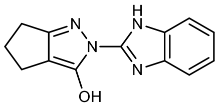

2-Hydrazinylbenzimidazole (1.00 equiv., 337 µmol, 50.0 mg, BLD BD75442) and methyl 2-oxocyclopentane-1-carboxylate (1.00 equiv, 337 µmol, 48.0 mg, BLD BD3822) were stirred in AcOH (1 mL) under reflux for 4 h. The reaction mixture was allowed to cool and concentrated in vacuum. The crude was purified by HPLC (method 1) to homogeneity, affording **10** (15%, 50.0 µmol, 11.9 mg).

^{1}H NMR (400 MHz, DMSO-d_6) δ 7.57 (dd, J = 6.0, 3.2 Hz, 2H), 7.29 (dd, J = 6.1, 3.6 Hz, 2H), 2.67 (t, J = 7.4 Hz, 2H), 2.54 – 2.46 (m, 2H), 2.33 (p, J = 7.4 Hz, 2H).

HRMS: Calc'd for $[M+H]^+$ 241.1084, found 241.1085.

2-(1*H*-Benzo[*d*]imidazol-2-yl)-4,5-dihydro-2*H*-benzo[*g*]indazol-3-ol (12)

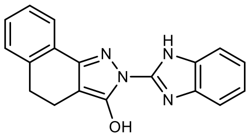

2-Hydrazinylbenzimidazole (1.00 equiv., 270 µmol, 40.0 mg) and methyl 1-oxo-1,2,3,4-tetrahydronaphthalene-2-carboxylate (1.00 equiv., 270 µmol, 55.1 mg, BLD BD335245) were stirred in AcOH (1 mL) under reflux for 3 h. The reaction mixture was allowed to cool and concentrated in vacuum. The crude was purified by HPLC (method 1) to homogeneity, affording **12** (4%, 11.1 µmol, 3.36 mg).

^{1}H NMR (400 MHz, DMSO-d_6) δ 7.86 – 7.82 (m, 1H), 7.57 (dd, J = 6.1, 3.3 Hz, 2H), 7.38 – 7.32 (m, 3H), 7.24 – 7.18 (m, 2H), 2.94 (t, J = 7.5 Hz, 2H), 2.60 (t, J = 7.4 Hz, 2H).

HRMS: Calc'd for $[M+H]^+$ 303.1241, found 303.1240.

1-(1*H*-Benzo[*d*]imidazol-2-yl)-4-benzyl-3-methyl-1*H*-pyrazol-5-ol (14)

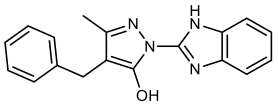

2-Hydrazinylbenzimidazole (1.00 equiv., 337 µmol, 50.0 mg) and ethyl 2-benzyl-3-oxobutanoate (1.00 equiv., 337 µmol, 74.3 mg, BLD BD128206) were reacted according to general method C. The crude was purified by HPLC (method 1) to homogeneity, affording **14** (69%, 232 µmol, 70.6 mg).

^{1}H NMR (400 MHz, DMSO-d_6) δ 7.57 – 7.48 (m, 2H), 7.31 – 7.23 (m, 4H), 7.20 – 7.12 (m, 3H), 3.60 (s, 2H), 2.16 (s, 3H).

HRMS: Calc'd for $[M+H]^+$ 305.1397, found 305.1397.

Methyl 2-(4-(((*tert*-butoxycarbonyl)amino)methyl)benzyl)-3-oxobutanoate (57)

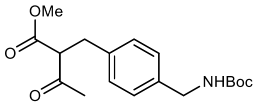

Methyl acetoacetate (1.00 equiv., 1.67 mmol, 179 µL, Sigma-Aldrich 537365) and *tert*-butyl (4-(bromomethyl)benzyl)carbamate (1.00 equiv., 1.67 mmol, 500 mg, BLD BD211549) were reacted according to general method B. The crude was purified by flash chromatography (Hex/EtOAc 5→50%) to afford **57** (71%, 1.18 mmol, 397 mg).

^{1}H NMR (400 MHz, $CDCl_3$) δ 7.18 (d, J = 8.1 Hz, 2H), 7.12 (d, J = 8.3 Hz, 2H), 4.81 (s, 1H), 4.26 (s, 2H), 3.76 (t, J = 7.6 Hz, 1H), 3.69 (s, 3H), 3.14 (d, J = 7.6 Hz, 2H), 2.18 (s, 3H), 1.45 (s, 9H).

HRMS: Calc'd for $[M+Na]^+$ 358.1625, found 358.1625.

4-(4-(Aminomethyl)benzyl)-1-(1*H*-benzo[*d*]imidazol-2-yl)-3-methyl-1*H*-pyrazol-5-ol (23)

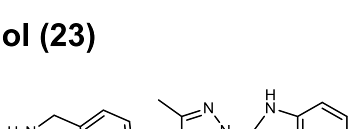

2-Hydrazinylbenzimidazole (1.20 equiv., 214 µmol, 31.7 mg) and **57** (1.00 equiv., 178 µmol, 59.7 mg) were stirred in EtOH (2 mL) at reflux for 2 h. The reaction mixture was allowed to cool and then concentrated in vacuum. The residue was purified by HPLC (method 1) to homogeneity. The isolated product was stirred in CF_3CO_2H (500 µL) at r.t. overnight. The reaction mixture was concentrated in vacuum to afford **23** as its trifluoroacetate salt (12% over two steps, 22.0 µmol, 9.84 mg) which was used without further purification.

^{1}H NMR (400 MHz, DMSO-d_6) δ 8.14 (s, 3H), 7.57 – 7.50 (m, 2H), 7.34 (q, *J* = 8.3 Hz, 4H), 7.19 (dq, *J* = 6.6, 3.5 Hz, 2H), 3.98 (q, *J* = 5.8 Hz, 2H), 3.63 (s, 2H), 2.16 (s, 3H).

^{19}F NMR (377 MHz, DMSO-d_6) δ -74.5 ($CF_3CO_2^-$).

HRMS: Calc'd for $C_{19}H_{20}N_5O^+$ 334.1662, found 334.1661. Calc'd for $CF_3CO_2^-$ 112.9856, found 112.9855.

1-(1*H*-Benzo[*d*]imidazol-2-yl)-4-(4-fluorobenzyl)-3-methyl-1*H*-pyrazol-5-ol (24)

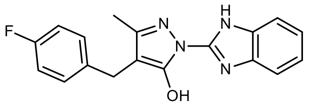

2-Hydrazinylbenzimidazole (1.10 equiv., 372 µmol, 55.2 mg) and ethyl 2-(4-fluorobenzyl)-3-oxobutanoate (1.00 equiv., 338 µmol, 80.6 mg, abcr AB526141) were reacted according to general method C. The crude was purified by HPLC (method 2) to homogeneity, affording **24** (77%, 260 µmol, 83.8 mg).

^{1}H NMR (400 MHz, DMSO-d_6) δ 7.52 (dt, *J* = 7.3, 3.6 Hz, 2H), 7.35 – 7.26 (m, 2H), 7.16 (dd, *J* = 6.0, 3.2 Hz, 2H), 7.12 – 7.05 (m, 2H), 3.58 (s, 2H), 2.16 (s, 3H).

^{19}F NMR (377 MHz, DMSO-d_6) δ -117.6.

HRMS: Calc'd for $[M+H]^+$ 323.1303, found 323.1301.

Methyl 3-hydroxy-2-(4-iodobenzyl)but-2-enoate (58)

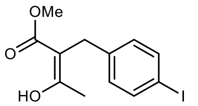

Methyl acetoacetate (1.20 equiv., 3.60 mmol, 387 µL) and 1-(bromomethyl)-4-iodobenzene (1.00 equiv., 3.00 mmol, 891 mg, BLD BD4513) were reacted according to general method B. The crude was purified by flash chromatography (Hex/EtOAc 0→30%) to afford **58** (14%, 420 µmol, 140 mg).

^{1}H NMR (400 MHz, DMSO-d_6) δ 8.33 (s, 1H), 7.58 (d, *J* = 8.3 Hz, 2H), 6.92 (d, *J* = 8.2 Hz, 2H), 3.49 (s, 3H), 3.45 (s, 2H), 1.85 (s, 3H).

HRMS: Calc'd for $[M+Na]^+$ 354.9802, found 354.9802.

1-(1*H*-Benzo[*d*]imidazol-2-yl)-4-(4-iodobenzyl)-3-methyl-1*H*-pyrazol-5-ol (27)

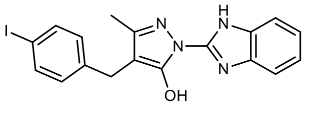

2-Hydrazinylbenzimidazole (1.10 equiv., 372 µmol, 55.2 mg) and **58** (1.00 equiv., 90.3 µmol, 30.0 mg) were reacted according to general method C. The crude was purified by HPLC (method 2) to homogeneity, affording **27** (70%, 63.6 µmol, 27.4 mg).

^{1}H NMR (400 MHz, DMSO-d_6) δ 7.62 (dd, *J* = 8.1, 1.6 Hz, 2H), 7.52 (dt, *J* = 7.2, 3.5 Hz, 2H), 7.16 (dt, *J* = 5.9, 3.6 Hz, 2H), 7.10 (d, *J* = 8.1 Hz, 2H), 3.55 (s, 2H), 2.15 (s, 3H).

HRMS: Calc'd for $[M+H]^+$ 431.0363, found 431.0362.

Methyl 3-amino-2-(4-(trifluoromethyl)benzyl)but-2-enoate (59)

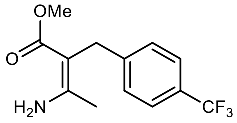

Methyl acetoacetate (1.20 equiv., 3.60 mmol, 387 µL) and 1-(bromomethyl)-4-(trifluoromethyl)benzene (1.00 equiv., 3.00 mmol, 717 mg, BLD BD33583) were reacted according to general method B. The crude was purified by flash chromatography (Hex/EtOAc 0→30%) to afford the enamine **59** (21%, 628 µmol, 172 mg) formed during aq. work-up.

^{1}H NMR (400 MHz, DMSO-d_6) δ 8.37 (s, 1H), 7.59 (d, *J* = 8.0 Hz, 2H), 7.32 (d, *J* = 8.0 Hz, 2H), 7.03 (s, 1H), 3.59 (s, 2H), 3.50 (s, 3H), 1.87 (s, 3H).

^{19}F NMR (377 MHz, DMSO-d_6) δ -60.6.

HRMS: Calc'd for $[M+H]^+$ 274.1049, found 274.1046.

1-(1*H*-Benzo[*d*]imidazol-2-yl)-3-methyl-4-(4-(trifluoromethyl)benzyl)-1*H*-pyrazol-5-ol (28)

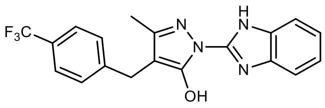

2-Hydrazinylbenzimidazole (1.10 equiv., 307 µmol, 45.5 mg) and **59** (1.00 equiv., 280 µmol, 76.6 mg) were reacted according to general method C. The crude was purified by HPLC (method 1) to homogeneity, affording **28** (47%, 131 µmol, 46.7 mg).

^{1}H NMR (400 MHz, DMSO-d_6) δ 7.63 (d, *J* = 8.0 Hz, 2H), 7.54 – 7.48 (m, 4H), 7.16 (dd, *J* = 6.0, 3.2 Hz, 2H), 3.69 (s, 2H), 2.18 (s, 3H).

^{19}F NMR (377 MHz, DMSO-d_6) δ -60.7.

HRMS: Calc'd for $[M+H]^+$ 373.1271, found 373.1270.

Methyl 2-(4-methoxybenzyl)-3-oxobutanoate (60)

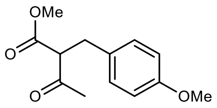

Methyl acetoacetate (1.20 equiv., 3.60 mmol, 387 µL) and 1-(bromomethyl)-4-methoxybenzene (1.00 equiv., 3.00 mmol, 603 mg, Apollo Scientific OR16607) were reacted according to general method B. The crude was purified by flash chromatography (Hex/EtOAc 0→30%) to afford **60** (81%, 2.43 mmol, 574 mg).

^{1}H NMR (400 MHz, $CDCl_3$) δ 7.08 (d, *J* = 8.6 Hz, 2H), 6.80 (d, *J* = 8.6 Hz, 2H), 3.77 (s, 3H), 3.74 (d, *J* = 7.6 Hz, 1H), 3.68 (s, 3H), 3.10 (d, *J* = 7.6 Hz, 2H), 2.16 (s, 3H).

HRMS: Calc'd for $[M+Na]^+$ 259.0941, found 249.0937.

1-(1*H*-Benzo[*d*]imidazol-2-yl)-4-(4-methoxybenzyl)-3-methyl-1*H*-pyrazol-5-ol (3)

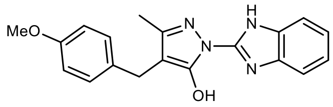

2-Hydrazinylbenzimidazole (1.10 equiv., 367 µmol, 54.4 mg) and **60** (1.00 equiv., 334 µmol, 78.8 mg) were reacted according to general method C. The crude was purified by HPLC (method 2) to homogeneity, affording **3** (84%, 282 µmol, 94.2 mg).

^{1}H NMR (500 MHz, DMSO-d_6, 353 K) δ 7.53 (dq, *J* = 6.7, 3.4 Hz, 2H), 7.22 – 7.13 (m, 4H), 6.86 – 6.81 (m, 2H), 3.72 (s, 3H), 3.56 (s, 2H), 2.15 (s, 3H).

^{13}C NMR (126 MHz, DMSO-d_6, 353 K) δ 159.8, 157.3, 150.1, 144.0, 136.2 (from HMBC), 132.3, 128.6, 121.3, 114.0, 113.5, 102.2, 54.8, 26.0, 11.0.

HRMS: Calc'd for $[M+H]^+$ 355.1503, found 355.1502.

3-(3-Oxo-1,3-dihydroisobenzofuran-1-yl)pentane-2,4-dione (61)

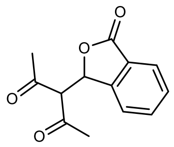

Methyl 2-formylbenzoate (1.00 equiv., 1.52 mmol, 208 µL, abcr AB134191), acetylacetone (1.10 equiv., 1.68 mmol, 172 µL, TCI P0052) and K_2CO_3 (0.30 equiv., 457 µmol, 63.14 mg) were stirred with a few drops of DMF at r.t. overnight. The reaction mixture was purified directly by flash chromatography (Hex/EtOAc 5→50%) to afford **61** as a single diastereomer (27%, 405 µmol, 94.1 mg).

^{1}H NMR (200 MHz, $CDCl_3$) δ 7.92 (dt, *J* = 7.2, 1.0 Hz, 1H), 7.75 – 7.48 (m, 2H), 7.38 (dd, *J* = 7.6, 1.2 Hz, 1H), 6.18 (d, *J* = 9.4 Hz, 1H), 4.00 (d, *J* = 9.4 Hz, 1H), 2.40 (s, 3H), 2.24 (s, 3H).

HRMS: Calc'd for $[M+Na]^+$ 255.0628, found 255.0629.

3-(1-(1*H*-Benzo[*d*]imidazol-2-yl)-3,5-dimethyl-1*H*-pyrazol-4-yl)isobenzofuran-1(3*H*)-one (29)

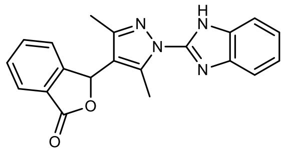

2-Hydrazinylbenzimidazole (1.20 equiv., 258 µmol, 38.3 mg) and **61** (1.00 equiv., 215 µmol, 50.0 mg) were reacted according to general method C. The crude was purified by HPLC (method 1) to homogeneity, affording **29** (quant., 215 µmol, 74.1 mg).

^{1}H NMR (400 MHz, $CDCl_3$) δ 8.00 (d, *J* = 7.5 Hz, 1H), 7.71 (t, 1H), 7.61 (t, *J* = 7.5 Hz, 1H), 7.57 – 7.49 (m, 2H), 7.35 (d, *J* = 7.6 Hz, 1H), 7.25 (dq, *J* = 7.1, 3.6 Hz, 2H), 6.49 (s, 1H), 2.79 (s, 3H), 1.83 (s, 3H).

HRMS: Calc'd for $[M+H]^+$ 345.1346, found 345.1346.

3-Oxo-2-(3-oxo-1,3-dihydroisobenzofuran-1-yl)butanenitrile (62)

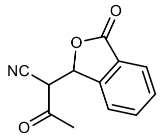

2-Formylbenzoic acid (1.00 equiv., 13.3 mmol, 2.00 g, BLD BD17732), 3-oxobutanenitrile (1.20 equiv., 16.0 mmol, 1.33 mL, BLD BD88750) and sulfamic acid (0.20 equiv., 2.66 mmol, 259 mg) were heated neat to 120 °C in a pressure tube for 2 h. The reaction mixture was allowed to cool, diluted with H_2O (15 mL) and extracted with CH_2Cl_2 (3 × 15 mL). The combined org. phases were dried over Na_2SO_4 and concentrated in vacuum. The crude was purified by flash chromatography (Hex/EtOAc 20→100%) to afford **62** as a mixture of diastereomers (35%, 4.72 mmol, 1.02 g).

^{1}H NMR (400 MHz, $CDCl_3$) δ 7.98 (dd, *J* = 9.8, 7.5 Hz, 1H), 7.80 – 7.71 (m, 1H), 7.65 (td, *J* = 7.5, 3.6 Hz, 1H), 7.57 (d, *J* = 7.7 Hz, 1H minor), 7.53 (d, *J* = 7.7 Hz, 1H major), 6.00 (d, *J* = 1.6 Hz, 1H major), 5.99 (s, 1H minor), 4.20 (d, *J* = 5.6 Hz, 1H minor), 4.01 (d, *J* = 3.1 Hz, 1H major), 2.56 (s, 3H major), 2.53 (s, 3H minor).

HRMS: Calc'd for $[M+H]^+$ 238.0475, found 238.0474.

3-(5-Amino-1-(1*H*-benzo[*d*]imidazol-2-yl)-3-methyl-1*H*-pyrazol-4-yl)isobenzofuran-1(3*H*)-one (30)

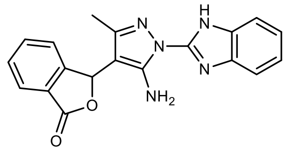

2-Hydrazinylbenzimidazole (1.10 equiv., 324 µmol, 48.0 mg) and **62** (1.00 equiv., 294 µmol, 63.4 mg) were reacted according to general method C. The crude was purified by HPLC (method 2) to homogeneity, affording **30** (53%, 156 µmol, 53.8 mg).

^{1}H NMR (400 MHz, DMSO-d_6) δ 7.94 (d, *J* = 7.6 Hz, 1H), 7.81 (td, *J* = 7.5, 1.1 Hz, 1H), 7.67 (t, *J* = 7.5 Hz, 1H), 7.55 (d, *J* = 7.6 Hz, 1H), 7.31 – 7.27 (m, 2H), 7.22 – 7.12 (m, 2H), 6.89 (s, 1H), 1.49 (s, 3H).

HRMS: Calc'd for $[M+H]^+$ 346.1299, found 346.1298.

Ethyl 3-cyclopropyl-2-(4-methoxybenzyl)-3-oxopropanoate (63)

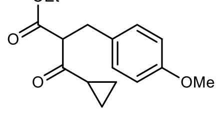

Ethyl 3-cyclopropyl-3-oxopropanoate (1.20 equiv., 3.42 mmol, 503 µL, BLD BD10708) and 1-(bromomethyl)-4-methoxybenzene (1.00 equiv., 2.85 mmol, 400 µL) were reacted according to general method B. The crude was purified by flash chromatography (Hex/EtOAc 0→50%) to afford **63** (91%, 2.58 mmol, 714 mg).

^{1}H NMR (400 MHz, $CDCl_3$) δ 7.13 – 7.08 (m, 2H), 6.83 – 6.77 (m, 2H), 4.23 – 4.08 (m, 2H), 3.86 (t, *J* = 7.6 Hz, 1H), 3.77 (s, 3H), 3.14 (d, *J* = 7.6 Hz, 2H), 2.04 (tt, *J* = 7.7, 4.5 Hz, 1H), 1.21 (t, *J* = 7.1 Hz, 3H), 1.08 – 0.99 (m, 2H), 0.94 – 0.85 (m, 2H).

HRMS: Calc'd for $[M+Na]^+$ 299.1254, found 299.1254.

1-(1*H*-Benzo[*d*]imidazol-2-yl)-3-cyclopropyl-4-(4-methoxybenzyl)-1*H*-pyrazol-5-ol (31)

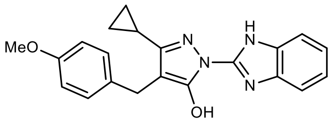

2-Hydrazinylbenzimidazole (1.20 equiv., 352 µmol, 52.2 mg) and **63** (1.00 equiv., 293 µmol, 81.1 mg) were reacted according to general method C. The crude was purified by HPLC (method 1) to homogeneity, affording **31** (82%, 239 µmol, 86.2 mg).

^{1}H NMR (400 MHz, DMSO-d_6) δ 7.51 (dt, *J* = 7.1, 3.6 Hz, 2H), 7.24 – 7.19 (m, 2H), 7.15 (dd, *J* = 6.0, 3.2 Hz, 2H), 6.88 – 6.80 (m, 2H), 3.70 (s, 3H), 3.62 (s, 2H), 2.05 – 1.91 (m, 1H), 1.04 – 0.89 (m, 4H).

HRMS: Calc'd for $[M+H]^+$ 361.1659, found 361.1659.

Ethyl 2-(4-methoxybenzyl)-3-oxo-3-(thiophen-2-yl)propanoate (64)

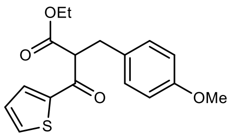

Ethyl 3-oxo-3-(thiophen-2-yl)propanoate (1.20 equiv., 3.42 mmol, 557 µL, BLD BD6708) and 1-(bromomethyl)-4-methoxybenzene (1.00 equiv., 2.85 mmol, 400 µL) were reacted according to general method B. The crude was purified by flash chromatography (Hex/EtOAc 0→50%) to afford **64** (quant., 2.85 mmol, 907 mg).

^{1}H NMR (400 MHz, $CDCl_3$) δ 7.75 (dd, *J* = 3.9, 1.1 Hz, 1H), 7.66 (dd, *J* = 4.9, 1.1 Hz, 1H), 7.16 – 7.12 (m, 2H), 7.10 (dd, *J* = 4.9, 3.9 Hz, 1H), 6.81 – 6.76 (m, 2H), 4.40 (t, *J* = 7.4 Hz, 1H), 4.12 (qd, *J* = 7.2, 2.1 Hz, 2H), 3.76 (s, 3H), 3.34 – 3.20 (m, 2H), 1.15 (t, *J* = 7.1 Hz, 3H).

HRMS: Calc'd for $[M+Na]^+$ 341.0818, found 341.0819.

1-(1*H*-Benzo[*d*]imidazol-2-yl)-4-(4-methoxybenzyl)-3-(thiophen-2-yl)-1*H*-pyrazol-5-ol (32)

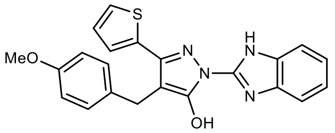

2-Hydrazinylbenzimidazole (1.20 equiv., 266 µmol, 39.4 mg) and **64** (1.00 equiv., 221 µmol, 70.5 mg) were reacted according to general method C. The crude was purified by HPLC (method 2) to homogeneity, affording **32** (15%, 32.8 µmol, 13.2 mg).

^{1}H NMR (400 MHz, DMSO-d_6) δ 7.66 – 7.55 (m, 3H), 7.34 (dd, *J* = 3.7, 1.2 Hz, 1H), 7.26 (dd, *J* = 6.0, 3.2 Hz, 2H), 7.20 – 7.15 (m, 2H), 7.15 – 7.10 (m, 1H), 6.87 – 6.81 (m, 2H), 3.85 (s, 2H), 3.69 (s, 3H).

HRMS: Calc'd for $[M+H]^+$ 403.1223, found 403.1224.

Ethyl 2-(4-methoxybenzyl)-3-oxo-3-(tetrahydro-2*H*-pyran-4-yl)propanoate (65)

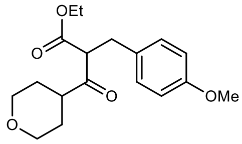

Ethyl 3-oxo-3-(tetrahydro-2H-pyran-4-yl)propanoate (1.20 equiv., 3.42 mmol, 610 µL, BLD BD211109) and 1-(bromomethyl)-4-methoxybenzene (1.00 equiv., 2.85 mmol, 400 µL) were reacted according to general method B. The crude was purified by flash chromatography (Hex/EtOAc 0→50%) to afford **65** (87%, 2.48 mmol, 793 mg).

^{1}H NMR (400 MHz, $CDCl_3$) δ 7.09 – 7.04 (m, 2H), 6.82 – 6.77 (m, 2H), 4.14 (qd, *J* = 7.1, 2.8 Hz, 2H), 3.98 – 3.83 (m, 3H), 3.77 (s, 3H), 3.47 – 3.17 (m, 3H), 3.10 (t, *J* = 7.2 Hz, 1H), 2.61 – 2.49 (m, 1H), 1.84 – 1.59 (m, 4H), 1.21 (t, *J* = 7.1 Hz, 3H).

HRMS: Calc'd for $[M+H]^+$ 343.1516, found 343.1517.

1-(1*H*-Benzo[*d*]imidazol-2-yl)-4-(4-methoxybenzyl)-3-(tetrahydro-2*H*-pyran-4-yl)-1*H*-pyrazol-5-ol (4)

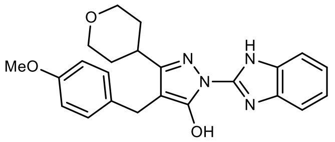

2-Hydrazinylbenzimidazole (1.20 equiv., 280 µmol, 41.5 mg) and **65** (1.00 equiv., 233 µmol, 74.8 mg) were reacted according to general method C. The crude was purified by HPLC (method 1) to homogeneity, affording **4** (27%, 62.3 µmol, 25.2 mg).

^{1}H NMR (400 MHz, DMSO-d_6) δ 7.53 (dt, *J* = 7.2, 3.6 Hz, 2H), 7.18 (dt, *J* = 5.9, 3.3 Hz, 4H), 6.87 – 6.80 (m, 2H), 3.93 – 3.85 (m, 2H), 3.70 (s, 3H), 3.60 (s, 2H), 3.36 (td, *J* = 11.8, 2.0 Hz, 2H), 2.96 (dd, *J* = 13.7, 10.1 Hz, 1H), 1.91 – 1.76 (m, 2H), 1.52 (dd, *J* = 12.2, 3.4 Hz, 2H).

^{13}C NMR (151 MHz, DMSO-d_6) δ 163.0, 157.5, 156.8, 144.3, 136.3, 132.8, 129.0, 121.8, 114.5, 113.7, 101.3, 66.9, 55.0, 33.2, 30.4, 26.1.

HRMS: Calc'd for $[M+H]^+$ 405.1921, found 405.1922.

Methyl 3-oxo-2-(3-oxo-1,3-dihydroisobenzofuran-1-yl)butanoate (66)

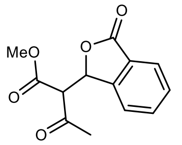

Methyl 2-formylbenzoate (1.00 equiv., 12.2 mmol, 1.67 mL), methyl acetoacetate (1.10 equiv., 13.4 mmol, 1.44 mL) and K_2CO_3 (0.30 equiv., 3.65 mmol, 505 mg) were stirred neat at r.t. overnight. The reaction mixture was adsorbed onto SiO_2 and purified by flash chromatography (Hex/EtOAc 6→60%) to afford **66** as a mixture of diastereomers (70%, 8.48 mmol, 2.11 g).

^{1}H NMR (400 MHz, $CDCl_3$) δ 7.90 (d, *J* = 7.5 Hz, 1H), 7.65 (tdt, *J* = 7.0, 4.6, 1.2 Hz, 1H), 7.58 – 7.53 (m, 1H), 7.48 (dd, *J* = 21.0, 7.7 Hz, 1H), 6.10 (d, *J* = 8.3 Hz, 1H), 3.89 (ddd, *J* = 8.3, 2.9, 1.3 Hz, 1H), 3.80 (s, 3H minor), 3.73 (s, 3H major), 2.38 (s, 3H major), 2.28 (s, 3H minor).

HRMS: Calc'd for $[M+Na]^+$ 271.0577, found 271.0579.

3-(1-(4,5-Dihydro-1*H*-imidazol-2-yl)-5-hydroxy-3-methyl-1*H*-pyrazol-4-yl)isobenzofuran-1(3*H*)-one (33)

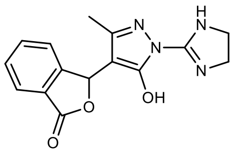

2-Hydrazinyl-4,5-dihydro-1*H*-imidazole hydrobromide (1.05 equiv., 338 µmol, 61.26 mg, BLD BD75443) and **66** (1.00 equiv., 322 µmol, 80.0 mg) were stirred in AcOH (1 mL) at r.t. overnight, then at 110 °C for 40 min. The reaction mixture was allowed to cool and concentrated in vacuum. The crude was purified by HPLC (method 1) to homogeneity, affording **33** (23%, 74.0 µmol, 22.1 mg).

^{1}H NMR (400 MHz, DMSO-d_6) δ 9.12 (s, 2H), 7.80 (d, *J* = 7.6 Hz, 1H), 7.71 (td, *J* = 7.5, 1.1 Hz, 1H), 7.55 (t, *J* = 7.5 Hz, 1H), 7.40 (d, *J* = 7.6 Hz, 1H), 6.40 (s, 1H), 3.69 (s, 4H), 1.60 (s, 3H).

HRMS: Calc'd for $[M+H]^+$ 299.1139, found 299.1138.

3-(5-Hydroxy-3-methyl-1-(1-methyl-1*H*-benzo[*d*]imidazol-2-yl)-1*H*-pyrazol-4-yl)isobenzofuran-1(3*H*)-one (35)

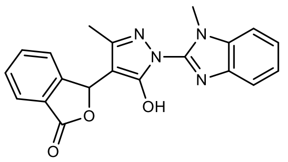

2-Hydrazinyl-1-methyl-1*H*-benzo[*d*]imidazole (1.05 equiv., 338 µmol, 54.9 mg, Enamine EN300-03618) and **66** (1.00 equiv., 322 µmol, 80.0 mg) were reacted according to general method C. The crude was purified by HPLC (method 1) to homogeneity, affording **35** (4%, 14.4 µmol, 5.20 mg).

^{1}H NMR (400 MHz, $CDCl_3$) δ 7.96 (d, *J* = 7.7 Hz, 1H), 7.68 (t, *J* = 7.4 Hz, 1H), 7.62 – 7.52 (m, 2H), 7.43 (d, *J* = 7.7 Hz, 1H), 7.41 – 7.33 (m, 3H), 6.56 (s, 1H), 4.28 (s, 3H), 1.85 (s, 3H).

HRMS: Calc'd for $[M+H]^+$ 361.1295, found 361.1295.

3-(5-Hydroxy-3-methyl-1-(quinazolin-2-yl)-1*H*-pyrazol-4-yl)isobenzofuran-1(3*H*)-one (36)

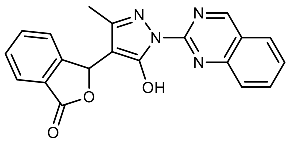

2-Hydrazinylquinazoline (1.00 equiv., 281 µmol, 45.0 mg, Enamine EN300-157628) and **66** (1.00 equiv., 281 µmol, 69.7 mg) were reacted according to general method C. The crude was purified by HPLC (method 1) to homogeneity, affording **36** (15%, 40.8 µmol, 14.6 mg).

^{1}H NMR (400 MHz, $CDCl_3$) δ 9.48 (s, 1H), 8.03 – 7.91 (m, 4H), 7.73 – 7.40 (m, 4H), 6.53 (s, 1H), 1.96 (s, 3H).

HRMS: Calc'd for $[M+H]^+$ 359.1139, found 359.1138.

3-(5-Hydroxy-3-methyl-1-(quinoxalin-2-yl)-1*H*-pyrazol-4-yl)isobenzofuran-1(3*H*)-one (37)

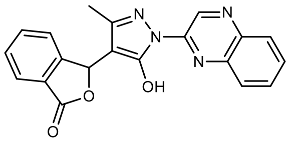

2-Hydrazinylquinoxaline (1.00 equiv., 281 µmol, 45.0 mg, Enamine EN300-66293) and **66** (1.00 equiv., 281 µmol, 69.7 mg) were reacted according to general method C. The crude was purified by HPLC (method 1) to afford **37** (67%, 189 µmol. 67.7 mg).

^{1}H NMR (400 MHz, $CDCl_3$) δ 9.58 (s, 1H), 8.14 (d, *J* = 8.1 Hz, 1H), 7.99 (d, *J* = 7.6 Hz, 1H), 7.88 – 7.67 (m, 4H), 7.60 (t, *J* = 7.5 Hz, 1H), 7.46 (d, *J* = 7.6 Hz, 1H), 6.53 (s, 1H), 1.96 (s, 3H).

HRMS: Calc'd for $[M+H]^+$ 359.1139, found 359.1138.

3-(1-(Benzo[*d*]oxazol-2-yl)-5-hydroxy-3-methyl-1*H*-pyrazol-4-yl)isobenzofuran-1(3*H*)-one (38)

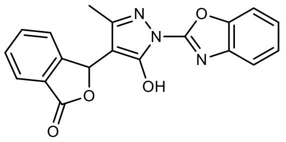

2-Hydrazinylbenzo[*d*]oxazole (1.00 equiv., 469 µmol, 70.0 mg, BLD BD27599) and **66** (1.00 equiv., 469 µmol, 117 mg) were stirred in CF_3CO_2H (500 µL) at r.t. overnight. The reaction mixture was concentrated in vacuum, and the crude was purified by HPLC (method 1) to homogeneity, affording **38** (4%, 20.6 µmol, 7.61 mg).

^{1}H NMR (400 MHz, $CDCl_3$) δ 7.99 (d, *J* = 7.7 Hz, 1H), 7.71 (td, *J* = 7.5, 1.1 Hz, 1H), 7.64 – 7.57 (m, 3H), 7.46 – 7.33 (m, 3H), 6.52 (s, 1H), 1.95 (s, 3H).

HRMS: Calc'd for $[M+Na]^+$ 370.0798, found 370.0799.

3-(1-(Benzo[*d*]thiazol-2-yl)-5-hydroxy-3-methyl-1*H*-pyrazol-4-yl)isobenzofuran-1(3*H*)-one (39)

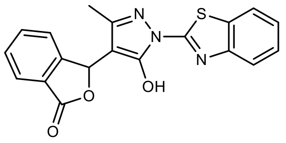

2-Hydrazinylbenzo[*d*]thiazole (1.05 equiv., 116 µmol, 19.1 mg, BLD BD4825) and **66** (1.00 equiv., 111 µmol, 27.5 mg) were stirred in AcOH (500 µL) at r.t. overnight, then under reflux for 30 min. The reaction mixture was allowed to cool and concentrated in vacuum. The crude was purified by HPLC (method 1) to homogeneity, affording **39** (99%, 110 µmol, 39.9 mg).

^{1}H NMR (400 MHz, $CDCl_3$) δ 7.97 (d, *J* = 7.6 Hz, 1H), 7.81 (d, *J* = 8.0 Hz, 1H), 7.77 – 7.65 (m, 2H), 7.58 (t, *J* = 7.5 Hz, 1H), 7.45 (d, *J* = 7.7 Hz, 2H), 7.39 – 7.31 (m, 1H), 6.51 (s, 1H), 1.92 (s, 3H).

HRMS: Calc'd for $[M+Na]^+$ 386.0570, found 386.0571.

Methyl 3-oxo-2-(3-oxoisoindolin-1-yl)butanoate (67)

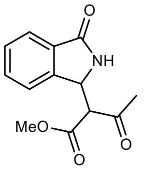

2-Formylbenzonitrile (1.00 equiv., 2.29 mmol, 300 mg, BLD BD22471) was added to a mixture of methyl acetoacetate (1.20 equiv., 2.75 mmol, 295 µL) and $CoCl_2$ (0.40 equiv., 915 µmol, 119 mg) in MeCN (11.4 mL). The reaction mixture was stirred at r.t. overnight, diluted with H_2O (20 mL) and extracted with EtOAc (3 × 20 mL). The combined org. phases were dried over Na_2SO_4 and concentrated in vacuum. The crude was purified by flash chromatography (Hex/EtOAc 10→100%) to afford **67** as a mixture of diastereomers (25%, 581 µmol, 143 mg).

^{1}H NMR (400 MHz, $CDCl_3$) δ 7.86 (ddt, *J* = 7.1, 2.1, 1.0 Hz, 1H), 7.60 – 7.45 (m, 2H), 7.32 – 7.27 (m, 1H), 6.86 (s, 1H major), 6.78 (s, 1H minor), 5.25 (t, *J* = 8.3 Hz, 1H), 3.87 (s, 3H minor), 3.78 (d, *J* = 8.1 Hz, 1H major), 3.71 (s, 3H major), 3.68 (d, *J* = 8.4 Hz, 1H minor), 2.35 (s, 3H major), 2.18 (s, 3H minor).

HRMS: Calc'd for $[M+H]^+$ 248.0917, found 248.0918.

3-(1-(1*H*-Benzo[*d*]imidazol-2-yl)-5-hydroxy-3-methyl-1*H*-pyrazol-4-yl)isoindolin-1-one (40)

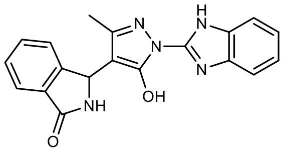

2-Hydrazinylbenzimidazole (1.20 equiv., 281 mmol, 41.6 mg) and **67** (1.00 equiv., 234 µmol, 57.9 mg) were reacted according to general method C. The crude was purified by HPLC (method 1) to homogeneity, affording **40** (18%, 42.9 µmol, 14.8 mg).

^{1}H NMR (400 MHz, DMSO-d_6) δ 8.69 (s, 1H), 7.68 (d, *J* = 7.5 Hz, 1H), 7.59 – 7.44 (m, 5H), 7.41 (d, *J* = 7.5 Hz, 1H), 7.19 (dd, *J* = 6.0, 3.2 Hz, 2H), 5.60 (s, 1H), 1.88 (s, 3H).

HRMS: Calc'd for $[M+H]^+$ 346.1299, found 346.1299.

Methyl 2-(5-chloro-3-oxo-1,3-dihydroisobenzofuran-1-yl)-3-oxobutanoate (68)

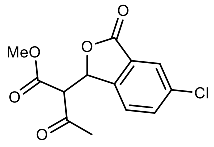

Methyl 5-chloro-2-formylbenzoate (1.00 equiv., 504 µmol, 100 mg, BLD BD00765001), methyl acetoacetate (1.10 equiv., 554 µmol, 59.6 µL) and K_2CO_3 (0.30 equiv., 151 µmol, 20.9 mg) were stirred with a few drops of DMF at 50 °C overnight. The reaction mixture was purified directly by flash chromatography (Hex/EtOAc 7→70%) to afford **68** as a mixture of diastereomers (35%, 174 µmol, 49.3 mg).

^{1}H NMR (200 MHz, $CDCl_3$) δ 7.83 (dq, *J* = 2.1, 0.7 Hz, 1H), 7.68 – 7.35 (m, 2H), 6.05 (d, *J* = 8.1 Hz, 1H), 3.94 (d, *J* = 4.4 Hz, 1H minor), 3.90 (d, *J* = 4.5 Hz, 1H major), 3.80 (s, 3H minor), 3.71 (s, 3H major), 2.37 (s, 3H major), 2.27 (s, 3H minor).

HRMS: Calc'd for $[M+Na]^+$ 305.0187, found 305.0187.

3-(1-(1*H*-Benzo[*d*]imidazol-2-yl)-5-hydroxy-3-methyl-1*H*-pyrazol-4-yl)-6-chloroisobenzofuran-1(3*H*)-one (41)

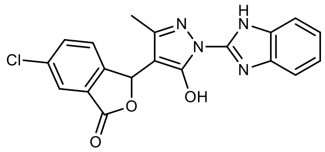

2-Hydrazinylbenzimidazole (1.20 equiv., 209 mmol, 31.0 mg) and **68** (1.00 equiv., 174 µmol, 49.3 mg) were reacted according to general method C. The crude was purified by HPLC (method 1) to homogeneity, affording **41** (54%, 93.7 µmol, 35.7 mg).

^{1}H NMR (400 MHz, DMSO-d_6) δ 7.87 (d, *J* = 2.0 Hz, 1H), 7.77 (dd, *J* = 8.2, 2.1 Hz, 1H), 7.59 – 7.44 (m, 3H), 7.27 (dq, *J* = 6.9, 3.9 Hz, 2H), 6.56 (s, 1H), 2.02 (s, 3H).

HRMS: Calc'd for $[M+H]^+$ 381.0749, found 381.0748.

Methyl 2-(6-chloro-3-oxo-1,3-dihydroisobenzofuran-1-yl)-3-oxobutanoate (69)

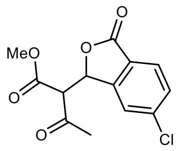

Methyl 4-chloro-2-formylbenzoate (1.00 equiv., 1.26 mmol, 250 mg, BLD BD00765914), methyl acetoacetate (1.10 equiv., 1.38 mmol, 149 µL) and K_2CO_3 (0.30 equiv., 378 µmol, 52.2 mg) were stirred with a few drops of DMF at 80 °C overnight. The reaction mixture was purified directly by flash chromatography (Hex/EtOAc 5→50%) to afford **69** as a mixture of diastereomers (9%, 115 µmol, 32.5 mg).

^{1}H NMR (400 MHz, $CDCl_3$) δ 7.81 (dt, *J* = 8.6, 2.3 Hz, 1H), 7.56 – 7.46 (m, 2H), 6.04 (d, *J* = 8.2 Hz, 1H), 3.96 – 3.91 (m, 1H major), 3.89 (d, *J* = 9.0 Hz, 1H minor), 3.82 (s, 3H minor), 3.74 (s, 3H major), 2.39 (s, 3H major), 2.29 (s, 3H minor).

HRMS: Calc'd for $[M+Na]^+$ 305.0187, found 305.0187.

3-(1-(1*H*-Benzo[*d*]imidazol-2-yl)-5-hydroxy-3-methyl-1*H*-pyrazol-4-yl)-5-chloroisobenzofuran-1(3*H*)-one (42)

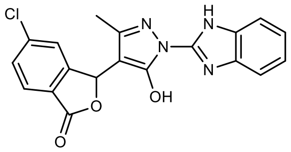

2-Hydrazinylbenzimidazole (1.20 equiv., 138 mmol, 20.4 mg) and **69** (1.00 equiv., 115 µmol, 32.5 mg) were reacted according to general method C. The crude was purified by HPLC (method 1) to homogeneity, affording **42** (26%, 29.5 µmol, 11.2 mg).

^{1}H NMR (400 MHz, $CDCl_3$) δ 7.84 (d, *J* = 8.2 Hz, 1H), 7.50 (d, *J* = 8.2 Hz, 1H), 7.45 (s, 1H), 7.40 – 7.32 (m, 4H), 6.56 (s, 1H), 1.84 (s, 3H).

HRMS: Calc'd for $[M+H]^+$ 381.0749, found 381.0749.

5-Fluoro-2-hydrazinylbenzimidazole (70)

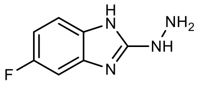

2-Chloro-5-fluorobenzimidazole (813 µmol, 139 mg, BLD BD73161) was reacted according to general method A. The crude was purified by flash chromatography (CH_2Cl_2/MeOH 0→30%) to afford **70** (83%, 677 µmol, 112 mg).

^{1}H NMR (400 MHz, DMSO-d_6) δ 7.94 (s, 1H), 7.05 (dd, *J* = 8.5, 5.0 Hz, 1H), 6.88 (dd, *J* = 9.9, 2.6 Hz, 1H), 6.64 (ddd, *J* = 10.7, 8.6, 2.6 Hz, 1H).

^{19}F NMR (377 MHz, DMSO-d_6) δ -124.5.

HRMS: Calc'd for $[M+H]^+$ 167.0728, found 167.0728.

3-(1-(5-Fluoro-1*H*-benzo[*d*]imidazol-2-yl)-5-hydroxy-3-methyl-1*H*-pyrazol-4-yl)isobenzofuran-1(3*H*)-one (43)

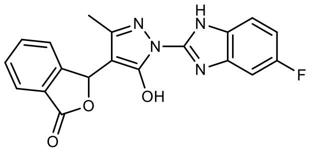

70 (1.02 equiv., 306 µmol, 50.8 mg) and **66** (1.00 equiv., 300 µmol 74.4 mg) were reacted according to general method C. The crude was purified by HPLC (method 1) to homogeneity, affording **43** (75%, 225 µmol, 82.1 mg).

^{1}H NMR (400 MHz, DMSO-d_6) δ 7.86 (d, *J* = 7.6 Hz, 1H), 7.74 (td, *J* = 7.5, 1.1 Hz, 1H), 7.59 (t, *J* = 7.5 Hz, 1H), 7.53 (d, *J* = 7.7 Hz, 1H), 7.48 (dd, *J* = 8.8, 4.8 Hz, 1H), 7.29 (dd, *J* = 9.1, 2.5 Hz, 1H), 7.06 (ddd, *J* = 9.8, 8.7, 2.6 Hz, 1H), 6.57 (s, 1H), 2.04 (s, 3H).

^{19}F NMR (377 MHz, DMSO-d_6) δ -119.9.

HRMS: Calc'd for $[M+H]^+$ 365.1044, found 365.1044.

5-Chloro-2-hydrazinylbenzimidazole (71)

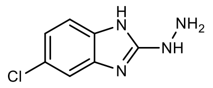

2,5-Dichlorobenzimidazole (559 µmol, 122 mg, Enamine EN300-697682) was reacted according to general method A. The crude was purified by flash chromatography (CH_2Cl_2/MeOH 0→50%) to afford **71** (89%, 497 µmol, 90.8 mg).

^{1}H NMR (400 MHz, DMSO-d_6) δ 8.02 (s, 1H), 7.10 (d, J = 2.2 Hz, 1H), 7.08 (d, J = 8.3 Hz, 1H), 6.84 (dd, J = 8.3, 2.1 Hz, 1H).

HRMS: Calc'd for $[M+H]^+$ 183.0432, found 183.0432.

3-(1-(5-Chloro-1*H*-benzo[*d*]imidazol-2-yl)-5-hydroxy-3-methyl-1*H*-pyrazol-4-yl)isobenzofuran-1(3*H*)-one (44)

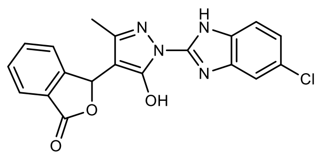

71 (1.00 equiv., 283 µmol, 51.7 mg) and **66** (1.04 equiv., 295 µmol, 73.2 mg) were reacted according to general method C. The crude was purified by HPLC (method 1) to homogeneity, affording **44** (67%, 189 µmol, 71.8 mg).

^{1}H NMR (400 MHz, DMSO-d_6) δ 7.86 (d, J = 7.6 Hz, 1H), 7.74 (td, J = 7.5, 1.1 Hz, 1H), 7.59 (t, J = 7.5 Hz, 1H), 7.56 – 7.46 (m, 3H), 7.22 (dd, J = 8.5, 2.0 Hz, 1H), 6.58 (s, 1H), 2.08 (s, 3H).

HRMS: Calc'd for $[M+H]^+$ 381.0749, found 381.0747.

2-Hydrazinyl-5-iodobenzimidazole (72)

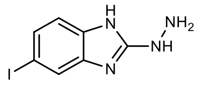

2-Chloro-5-iodobenzimidazole (407 µmol, 113 mg, BLD BD58603) was reacted according to general method A. The crude was purified by flash chromatography (CH_2Cl_2/MeOH 0→35%) to afford **72** (33%, 133 µmol, 36.4 mg).

^{1}H NMR (400 MHz, DMSO-d_6) δ 8.03 (s, 1H), 7.40 (d, J = 1.7 Hz, 1H), 7.13 (dd, J = 8.1, 1.8 Hz, 1H), 6.95 (d, J = 8.2 Hz, 1H).

HRMS: Calc'd for $[M+H]^+$ 274.9788, found 274.9789.

3-(5-Hydroxy-1-(5-iodo-1*H*-benzo[*d*]imidazol-2-yl)-3-methyl-1*H*-pyrazol-4-yl)isobenzofuran-1(3*H*)-one (45)

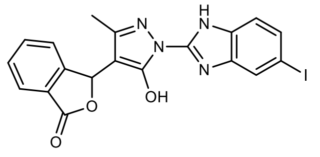

72 (1.00 equiv., 207 µmol, 56.6 mg) and **66** (1.14 equiv., 235 µmol, 58.4 mg) were reacted according to general method C. The crude was purified by HPLC (method 1) to homogeneity, affording **45** (76%, 157 µmol, 74.1 mg).

^1H NMR (400 MHz, DMSO-d_6) δ 7.85 (d, J = 7.6 Hz, 1H), 7.82 (d, J = 1.6 Hz, 1H), 7.74 (td, J = 7.5, 1.1 Hz, 1H), 7.59 (t, J = 7.5 Hz, 1H), 7.55 – 7.49 (m, 2H), 7.33 (d, J = 8.4 Hz, 1H), 6.57 (s, 1H), 2.06 (s, 3H).

HRMS: Calc'd for [M+H]+ 473.0105, found 473.0105.

2-Hydrazinyl-5-methylbenzimidazole (73)

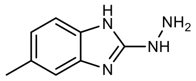

2-Chloro-5-methylbenzimidazole (848 µmol, 141 mg, BLD BD58619) was reacted according to general method A. The crude was purified by flash chromatography (CH_2Cl_2/MeOH 0→30%) to afford **73** (83%, 702 µmol, 114 mg).

^1H NMR (400 MHz, DMSO-d_6) δ 7.74 (s, 1H), 7.00 (d, J = 7.9 Hz, 1H), 6.94 (d, J = 1.6 Hz, 1H), 6.67 (dd, J = 7.8, 1.7 Hz, 1H), 2.30 (s, 3H).

HRMS: Calc'd for $[M+H]^+$ 163.0978, found 163.0979.

3-(5-Hydroxy-3-methyl-1-(5-methyl-1*H*-benzo[*d*]imidazol-2-yl)-1*H*-pyrazol-4-yl)isobenzofuran-1(3*H*)-one (46)

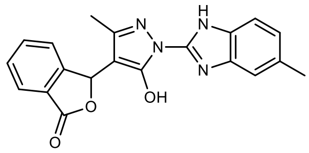

73 (1.06 equiv., 331 µmol, 53.4 mg) and **66** (1.00 equiv., 313 µmol, 77.8 mg) were reacted according to general method C. The crude was purified by HPLC (method 1) to homogeneity, affording **46** (75%, 235 µmol, 84.8 mg).

^1H NMR (400 MHz, DMSO-d_6) δ 7.84 (d, J = 7.6 Hz, 1H), 7.73 (td, J = 7.5, 1.1 Hz, 1H), 7.57 (t, J = 7.5 Hz, 1H), 7.49 (d, J = 7.6 Hz, 1H), 7.40 (d, J = 8.2 Hz, 1H), 7.32 (s, 1H), 7.10 (dd, J = 8.3, 1.6 Hz, 1H), 6.53 (s, 1H), 2.39 (s, 3H), 1.87 (s, 3H).

HRMS: Calc'd for $[M+H]^+$ 361.1295, found 361.1296.

2-Hydrazinyl-5-(trifluoromethyl)benzimidazole (74)

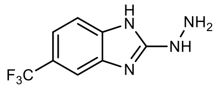

2-Chloro-5-(trifluoromethyl)benzimidazole (4.80 mmol, 1.06 g, BLD BD73103) was reacted according to general method A at 100 °C. The crude was purified by flash chromatography (CH_2Cl_2/MeOH 0→50%) to afford **74** (17%, 840 µmol, 182 mg).

^1H NMR (400 MHz, DMSO-d_6) δ 8.23 (s, 1H), 7.37 (d, J = 1.7 Hz, 1H), 7.24 (d, J = 8.2 Hz, 1H), 7.18 (dd, J = 8.3, 1.8 Hz, 1H).

^{19}F NMR (377 MHz, DMSO-d_6) δ -58.2.

HRMS: Calc'd for $[M+H]^+$ 217.0696, found 217.0697.

3-(5-Hydroxy-3-methyl-1-(5-(trifluoromethyl)-1*H*-benzo[*d*]imidazol-2-yl)-1*H*-pyrazol-4-yl)isobenzofuran-1(3*H*)-one (47)

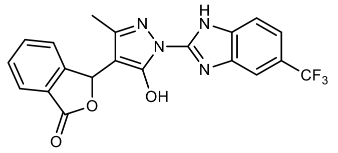

74 (1.00 equiv., 231 µmol, 50.0 mg) and **66** (1.10 equiv., 254 µmol, 63.2 mg) were reacted according to general method C. The crude was purified by HPLC (method 1) to homogeneity, affording **47** (86%, 198 µmol, 82.2 mg).

^{1}H NMR (400 MHz, DMSO-d_6) δ 7.87 (d, *J* = 7.6 Hz, 1H), 7.80 (d, *J* = 1.8 Hz, 1H), 7.75 (td, *J* = 7.5, 1.1 Hz, 1H), 7.67 (d, *J* = 8.4 Hz, 1H), 7.60 (t, *J* = 7.5 Hz, 1H), 7.55 (d, *J* = 7.6 Hz, 1H), 7.51 (dd, *J* = 8.6, 1.8 Hz, 1H), 6.60 (s, 1H), 2.14 (s, 3H).

^{19}F NMR (377 MHz, DMSO-d_6) δ -58.9.

HRMS: Calc'd for $[M+H]^+$ 415.1013, found 415.1013.

2-Hydrazinyl-5-methoxybenzimidazole (75)

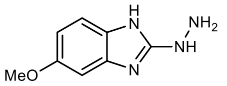

2-Chloro-5-methoxybenzimidazole (548 µmol, 100 mg, BLD BD6083) was reacted according to general method A. **75** was obtained as a mixture with hydrazinium chloride that was used without further purification (149 mg).

^{1}H NMR (400 MHz, DMSO-d_6) δ 7.00 (d, *J* = 8.4 Hz, 1H), 6.72 (d, *J* = 2.4 Hz, 1H), 6.46 (dd, *J* = 8.4, 2.5 Hz, 1H), 3.69 (s, 3H).

3-(5-Hydroxy-1-(5-methoxy-1*H*-benzo[*d*]imidazol-2-yl)-3-methyl-1*H*-pyrazol-4-yl)isobenzofuran-1(3*H*)-one (48)

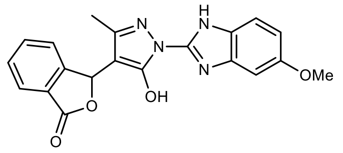

Crude **75** (70.0 mg) and **66** (255 µmol, 63.4 mg) were reacted according to general method C. The crude was purified by HPLC (method 1) to homogeneity, affording **48** (22% with respect to **66**, 56.3 µmol, 21.2 mg).

^{1}H NMR (400 MHz, DMSO-d_6) δ 7.84 (d, *J* = 7.6 Hz, 1H), 7.73 (td, *J* = 7.5, 1.0 Hz, 1H), 7.57 (t, *J* = 7.6 Hz, 1H), 7.50 (d, *J* = 7.7 Hz, 1H), 7.41 (d, *J* = 8.8 Hz, 1H), 7.04 (d, *J* = 2.4 Hz, 1H), 6.88 (dd, *J* = 8.8, 2.5 Hz, 1H), 6.53 (s, 1H), 3.77 (s, 3H), 1.88 (s, 3H).

HRMS: Calc'd for $[M+H]^+$ 377.1244, found 377.1243.

3-(5-Hydroxy-3-methyl-1-(5-nitro-1*H*-benzo[*d*]imidazol-2-yl)-1*H*-pyrazol-4-yl)isobenzofuran-1(3*H*)-one (5)

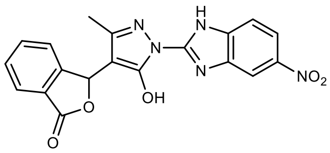

2-Hydrazinyl-5-nitrobenzimidazole (1.00 equiv., 278 µmol, 53.7 mg, abcr AB236804) and **66** (1.05 equiv., 293 µmol, 72.7 mg) were reacted according to general method C. The crude was purified by HPLC (method 1) to homogeneity, affording **5** (30%, 83.8 µmol, 32.8 mg).

^{1}H NMR (500 MHz, DMSO-d_6, 353 K) δ 8.35 (d, *J* = 2.3 Hz, 1H), 8.09 (dd, *J* = 8.8, 2.3 Hz, 1H), 7.87 (d, *J* = 7.6 Hz, 1H), 7.75 (td, *J* = 7.5, 1.1 Hz, 1H), 7.67 (d, *J* = 8.9 Hz, 1H), 7.61 (t, *J* = 7.5 Hz, 1H), 7.56 (d, *J* = 7.7 Hz, 1H), 6.59 (s, 1H), 2.15 (s, 3H).

^{13}C NMR (126 MHz, DMSO-d_6, 353 K) δ 169.4, 160.0, 152.4, 148.3, 145.9, 142.4, 141.5 (from HMBC), 135.8 (from HMBC), 133.7, 128.7, 125.9, 124.1, 122.7, 117.5, 114.2, 110.1, 96.6 (from HMBC), 74.3, 10.8.

HRMS: Calc'd for $[M+H]^+$ 392.0989, found 392.0989.

2-Hydrazinyl-4-fluorobenzimidazole (76)

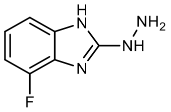

2-Chloro-4-fluorobenzimidazole (689 µmol, 117 mg, Fluorochem F077457) was reacted according to general method A. The crude was purified by flash chromatography (CH_2Cl_2/MeOH 0→25%) to afford **76** (86%, 590 µmol, 98.1 mg).

^{1}H NMR (400 MHz, DMSO-d_6) δ 11.23 (s, 1H), 7.95 (s, 1H), 6.97 (d, *J* = 7.7 Hz, 1H), 6.79 (td, *J* = 7.9, 4.9 Hz, 1H), 6.70 (dd, *J* = 11.1, 8.0 Hz, 1H), 4.54 (s, 2H).

^{19}F NMR (377 MHz, DMSO-d_6) δ -133.4.

HRMS: Calc'd for $[M+H]^+$ 167.0728, found 167.0727.

3-(1-(4-Fluoro-1*H*-benzo[*d*]imidazol-2-yl)-5-hydroxy-3-methyl-1*H*-pyrazol-4-yl)isobenzofuran-1(3*H*)-one (49)

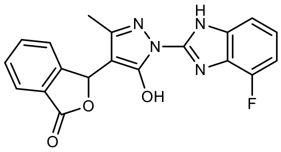

76 (1.00 equiv., 304 µmol, 50.1 mg) and **66** (1.21 equiv., 369 µmol, 91.2 mg) were reacted according to general method C. The crude was purified by HPLC (method 1) to homogeneity, affording **49** (88%, 268 µmol, 97.6 mg).

^{1}H NMR (400 MHz, DMSO-d_6) δ 7.87 (d, *J* = 7.7 Hz, 1H), 7.75 (td, *J* = 7.5, 1.1 Hz, 1H), 7.60 (t, *J* = 7.7 Hz, 1H), 7.56 (d, *J* = 7.7 Hz, 1H), 7.31 (d, *J* = 7.9 Hz, 1H), 7.11 (td, *J* = 8.0, 4.8 Hz, 1H), 6.99 (dd, *J* = 11.1, 8.1 Hz, 1H), 6.61 (s, 1H), 2.19 (s, 3H).

^{19}F NMR (377 MHz, DMSO-d_6) δ -130.3.

HRMS: Calc'd for $[M+H]^+$ 365.1044, found 365.1045.

4-Chloro-2-hydrazinylbenzimidazole (77)

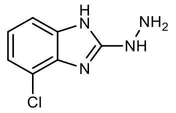

2,4-Dichlorobenzimidazole (556 µmol, 104 mg, BLD BD217434) was reacted according to general method A. The crude was purified by flash chromatography (CH_2Cl_2/MeOH 0→25%) to afford **77** (64%, 357 µmol, 65.3 mg).

^{1}H NMR (400 MHz, DMSO-d_6) δ 11.26 (s, 1H), 8.08 (s, 1H), 7.08 (dd, *J* = 7.7, 1.0 Hz, 1H), 6.93 (dd, *J* = 7.9, 1.0 Hz, 1H), 6.80 (t, *J* = 7.8 Hz, 1H), 4.55 (s, 2H).

HRMS: Calc'd for $[M+H]^+$ 183.0432, found 183.0432.

3-(1-(4-Chloro-1*H*-benzo[*d*]imidazol-2-yl)-5-hydroxy-3-methyl-1*H*-pyrazol-4-yl)isobenzofuran-1(3*H*)-one (50)

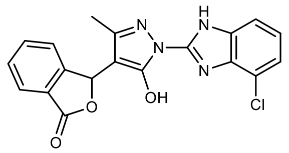

77 (1.00 equiv., 278 µmol, 50.7 mg) and **66** (1.39 equiv., 386 µmol, 95.8 mg) were reacted according to general method C. The crude was purified by HPLC (method 1) to homogeneity, affording **50** (70%, 195 µmol, 74.3 mg).

^{1}H NMR (400 MHz, DMSO-d_6) δ 7.87 (d, *J* = 7.6 Hz, 1H), 7.75 (td, *J* = 7.5, 1.1 Hz, 1H), 7.60 (t, *J* = 7.6 Hz, 1H), 7.56 (d, *J* = 7.7 Hz, 1H), 7.44 (dd, *J* = 8.0, 1.0 Hz, 1H), 7.25 – 7.20 (m, 1H), 7.13 (t, *J* = 7.9 Hz, 1H), 6.61 (s, 1H), 2.22 (s, 3H).

HRMS: Calc'd for $[M+H]^+$ 381.0749, found 381.0749.

2-Hydrazinyl-4-methylbenzimidazole (78)

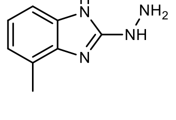

2-Chloro-4-methylbenzimidazole (604 µmol, 101 mg, BLD BD58617) was reacted according to general method A. The crude was purified by flash chromatography (CH_2Cl_2/MeOH 0→30%) to afford **78** (75%, 452 µmol, 73.3 mg).

^{1}H NMR (400 MHz, DMSO-d_6) δ 7.76 (s, 1H), 6.97 (d, *J* = 7.6 Hz, 1H), 6.76 (t, *J* = 7.5 Hz, 1H), 6.69 (d, *J* = 7.4 Hz, 1H), 2.36 (s, 3H).

HRMS: Calc'd for $[M+H]^+$ 163.0978, found 163.0978.

3-(5-Hydroxy-3-methyl-1-(4-methyl-1*H*-benzo[*d*]imidazol-2-yl)-1*H*-pyrazol-4-yl)isobenzofuran-1(3*H*)-one (51)

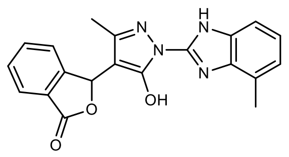

78 (1.03 equiv., 320 µmol, 51.8 mg) and **66** (1.00 equiv., 310 µmol, 77.0 mg) were reacted according to general method C. The crude was purified by HPLC (method 1) to homogeneity, affording **51** (74%, 231 µmol, 83.2 mg).

^{1}H NMR (400 MHz, DMSO-d_6) δ 7.85 (d, *J* = 7.6 Hz, 1H), 7.74 (td, *J* = 7.5, 1.1 Hz, 1H), 7.58 (t, *J* = 7.6 Hz, 1H), 7.51 (d, *J* = 7.6 Hz, 1H), 7.38 (d, *J* = 7.9 Hz, 1H), 7.14 (t, *J* = 7.7 Hz, 1H), 7.06 (d, *J* = 7.4 Hz, 1H), 6.55 (s, 1H), 2.52 (s, 3H), 1.97 (s, 3H).

HRMS: Calc'd for $[M+H]^+$ 361.1295, found 361.1295.

2-Hydrazinyl-4-(trifluoromethyl)benzimidazole (79)

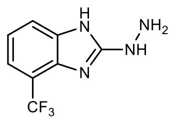

2-Chloro-4-(trifluoromethyl)benzimidazole (520 µmol, 115 mg, BLD BD303118) was reacted according to general method A. The crude was purified by flash chromatography (CH_2Cl_2/MeOH 0→30%) to afford **79** (43%, 222 µmol, 48.0 mg).

^{1}H NMR (400 MHz, DMSO-d_6) δ 11.41 (s, 1H), 8.31 (s, 1H), 7.35 (d, *J* = 7.7 Hz, 1H), 7.16 (d, *J* = 7.8 Hz, 1H), 6.92 (t, *J* = 7.8 Hz, 1H), 4.61 (s, 2H).

^{19}F NMR (377 MHz, DMSO-d_6) δ -59.3.

HRMS: Calc'd for $[M+H]^+$ 217.0696, found 217.0695.

3-(5-Hydroxy-3-methyl-1-(4-(trifluoromethyl)-1*H*-benzo[*d*]imidazol-2-yl)-1*H*-pyrazol-4-yl)isobenzofuran-1(3*H*)-one (52)

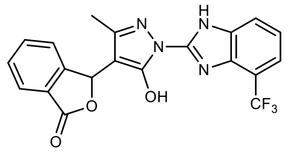

79 (1.00 equiv., 200 µmol, 43.3 mg) and **66** (1.10 equiv., 220 µmol, 54.6 mg) were reacted according to general method C. The crude was purified by HPLC (method 1) to homogeneity, affording **52** (76%, 152 µmol, 63.1 mg).

^{1}H NMR (400 MHz, DMSO-d_6) δ 7.88 (d, *J* = 7.6 Hz, 1H), 7.76 (t, *J* = 7.7 Hz, 2H), 7.64 – 7.54 (m, 2H), 7.49 (d, *J* = 7.7 Hz, 1H), 7.29 (t, *J* = 7.9 Hz, 1H), 6.63 (s, 1H), 2.24 (s, 3H).

^{19}F NMR (377 MHz, DMSO-d_6) δ -59.1.

HRMS: Calc'd for $[M+H]^+$ 415.1013, found 415.1014.

2-Hydrazinyl-4-methoxybenzimidazole (80)

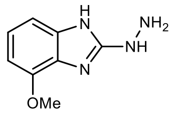

2-Chloro-4-methoxybenzimidazole (639 µmol, 117 mg, Fluorochem F614423) was reacted according to general method A. The crude was purified by flash chromatography (CH_2Cl_2/MeOH 0→30%) to afford **80** (88%, 560 µmol, 99.9 mg).

^1H NMR (400 MHz, DMSO-d_6) δ 7.60 (s, 1H), 6.83 – 6.75 (m, 2H), 6.51 (dd, J = 7.2, 1.8 Hz, 1H), 3.85 (s, 3H).

HRMS: Calc'd for $[M+H]^+$ 179.0927, found 179.0927.

3-(5-Hydroxy-1-(4-methoxy-1*H*-benzo[*d*]imidazol-2-yl)-3-methyl-1*H*-pyrazol-4-yl)isobenzofuran-1(3*H*)-one (53)

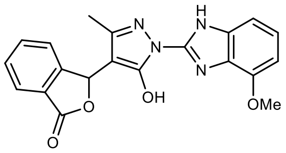

80 (1.00 equiv., 291 µmol, 51.8 mg) and **66** (1.09 equiv., 317 µmol, 78.7 mg) were reacted according to general method C. The crude was purified by HPLC (method 1) to homogeneity, affording **53** (21%, 60.5 µmol, 22.8 mg).

^1H NMR (400 MHz, DMSO-d_6) δ 7.86 (d, J = 7.6 Hz, 1H), 7.74 (td, J = 7.5, 1.1 Hz, 1H), 7.59 (t, J = 7.5 Hz, 1H), 7.54 (d, J = 7.6 Hz, 1H), 7.09 (d, J = 4.3 Hz, 2H), 6.80 – 6.71 (m, 1H), 6.58 (s, 1H), 3.90 (s, 3H), 2.08 (s, 3H).

HRMS: Calc'd for $[M+H]^+$ 377.1244, found 377.1245.

3-(1-(5,6-Difluoro-1*H*-benzo[*d*]imidazol-2-yl)-5-hydroxy-3-methyl-1*H*-pyrazol-4-yl)isobenzofuran-1(3*H*)-one (54)

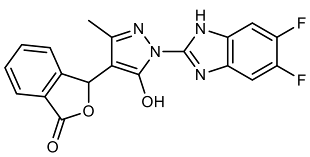

5,6-Difluoro-2-hydrazinylbenzimidazole (1.00 equiv., 103 µmol, 18.9 mg, Enamine EN300-28919) and **66** (1.20 equiv., 123 µmol, 30.6 mg) were reacted according to general method C. The crude was purified by HPLC (method 1) to homogeneity, affording **54** (45%, 45.9 µmol, 17.5 mg).

^1H NMR (400 MHz, $CDCl_3$) δ 7.91 (d, J = 7.6 Hz, 1H), 7.68 (t, J = 7.5 Hz, 1H), 7.54 (t, J = 7.5 Hz, 1H), 7.44 (d, J = 7.7 Hz, 1H), 7.18 (t, J = 8.2 Hz, 2H), 6.49 (s, 1H), 1.86 (s, 3H).

^{19}F NMR (377 MHz, $CDCl_3$) δ -136.8.

HRMS: Calc'd for $[M+Na]^+$ 405.0770, found 405.0770.

5,6-Dichloro-2-hydrazinylbenzimidazole (81)

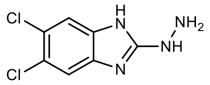

2,5,6-Trichlorobenzimidazole (1.17 mmol, 260 mg, BLD BD67291) was reacted according to general method A. The crude was purified by flash chromatography (CH_2Cl_2/MeOH 0→20%) to afford **81** (65%, 756 µmol, 164 mg).

^{1}H NMR (400 MHz, DMSO-d_6) δ 8.24 (s, 1H), 7.25 (s, 2H).

HRMS: Calc'd for $[M+H]^+$ 217.0042, found 217.0042.

3-(1-(5,6-Dichloro-1*H*-benzo[*d*]imidazol-2-yl)-5-hydroxy-3-methyl-1*H*-pyrazol-4-yl)isobenzofuran-1(3*H*)-one (55)

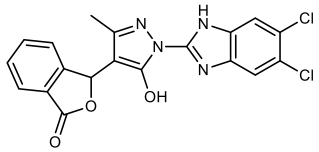

81 (1.00 equiv., 253 µmol, 55.0 mg) and **66** (1.10 equiv., 279 µmol, 69.2 mg) were reacted according to general method C. The crude was purified by HPLC (method 1) to homogeneity, affording **55** (75%, 191 µmol, 79.3 mg).

^{1}H NMR (400 MHz, DMSO-d_6) δ 7.86 (d, *J* = 7.6 Hz, 1H), 7.74 (td, *J* = 7.5, 1.1 Hz, 1H), 7.69 (s, 2H), 7.59 (t, *J* = 7.5 Hz, 1H), 7.55 (d, *J* = 7.6 Hz, 1H), 6.59 (s, 1H), 2.15 (s, 3H).

HRMS: Calc'd for $[M+H]^+$ 415.0359, found 415.0362.

4-(4-Methoxybenzyl)-3-methyl-1-(5-nitro-1*H*-benzo[*d*]imidazol-2-yl)-1*H*-pyrazol-5-ol (6)

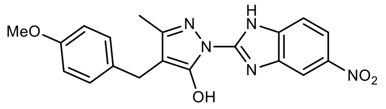

2-Hydrazinyl-5-nitrobenzimidazole (1.10 equiv., 287 µmol, 55.4 mg) and **60** (1.00 equiv., 261 µmol, 61.6 mg) were reacted according to general method C. The crude was purified by HPLC (method 2) to homogeneity, affording **6** (22%, 57.4 µmol, 21.8 mg).

1H NMR (500 MHz, DMSO-d_6, 353 K) δ 8.38 (d, *J* = 2.3 Hz, 1H), 8.09 (dd, *J* = 8.8, 2.3 Hz, 1H), 7.68 (d, *J* = 8.8 Hz, 1H), 7.19 (d, *J* = 8.6 Hz, 2H), 6.84 (d, *J* = 8.8 Hz, 2H), 3.72 (s, 3H), 3.56 (s, 2H), 2.18 (s, 3H).

^{13}C NMR (126 MHz, DMSO-d_6, 353 K) δ 161.2 (from HMBC), 157.4, 151.3, 146.8, 142.2, 136.0 (from HMBC), 132.1, 128.6, 117.3, 114.2, 113.6, 110.1, 103.1, 54.8, 26.0, 10.9. The signal of the carbon attached to the nitro group could not be detected by ^{13}C nor HMBC NMR at 298, 323 and 353 K due to intermediate exchange benzimidazole tautomerism[2].

HRMS: Calc'd for $[M+H]^+$ 380.1353, found 380.1353.

3-Methyl-1-(5-nitro-1*H*-benzo[*d*]imidazol-2-yl)-4-(4-(trifluoromethyl)benzyl)-1*H*-pyrazol-5-ol (56)

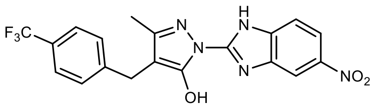

2-Hydrazinyl-5-nitrobenzimidazole (1.10 equiv., 268 μmol, 51.7 mg) and **59** (1.00 equiv., 243 μmol, 66.7 mg) were reacted according to general method C. The crude was purified by HPLC (method 2) to homogeneity, affording **56** (42%, 102 μmol, 42.6 mg).

^{1}H NMR (400 MHz, DMSO-d_6) δ 8.38 (s, 1H), 8.11 (dd, J = 8.9, 2.3 Hz, 1H), 7.68 (d, J = 8.9 Hz, 1H), 7.64 (d, J = 8.1 Hz, 2H), 7.51 (d, J = 8.0 Hz, 2H), 3.71 (s, 2H), 2.22 (s, 3H).

^{19}F NMR (377 MHz, DMSO-d_6) δ -60.7.

HRMS: Calc'd for $[M+H]^+$ 418.1122, found 418.1124.

NMR spectra

^{1}H and ^{13}C NMR spectra for all synthesized compounds depicted in the main text (Fig. 2a) are shown, as well as HMBC spectra to derive carbon signals broadened beyond detection in ^{13}C spectra.

1-(1*H*-Benzo[*d*]imidazol-2-yl)-4-(4-methoxybenzyl)-3-methyl-1*H*-pyrazol-5-ol (3)

^{1}H

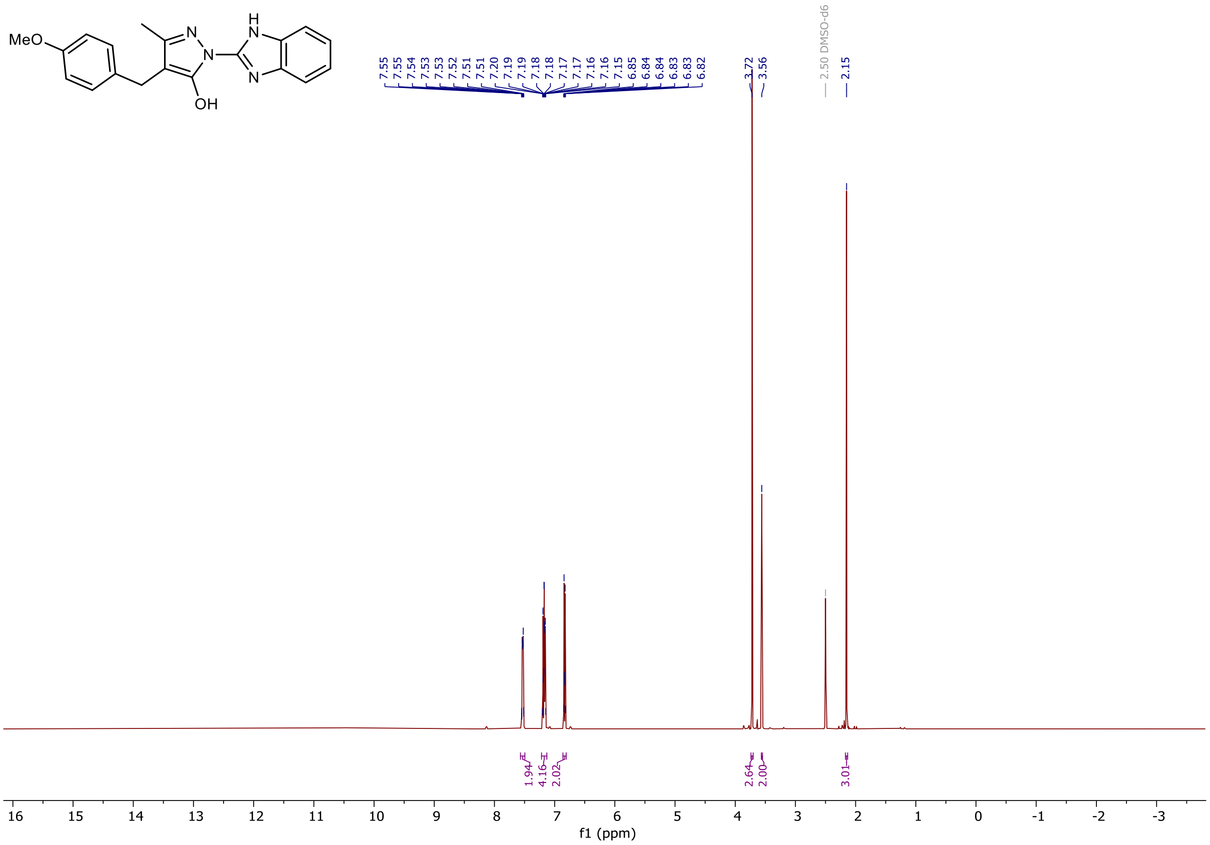

^{13}C DEPTq

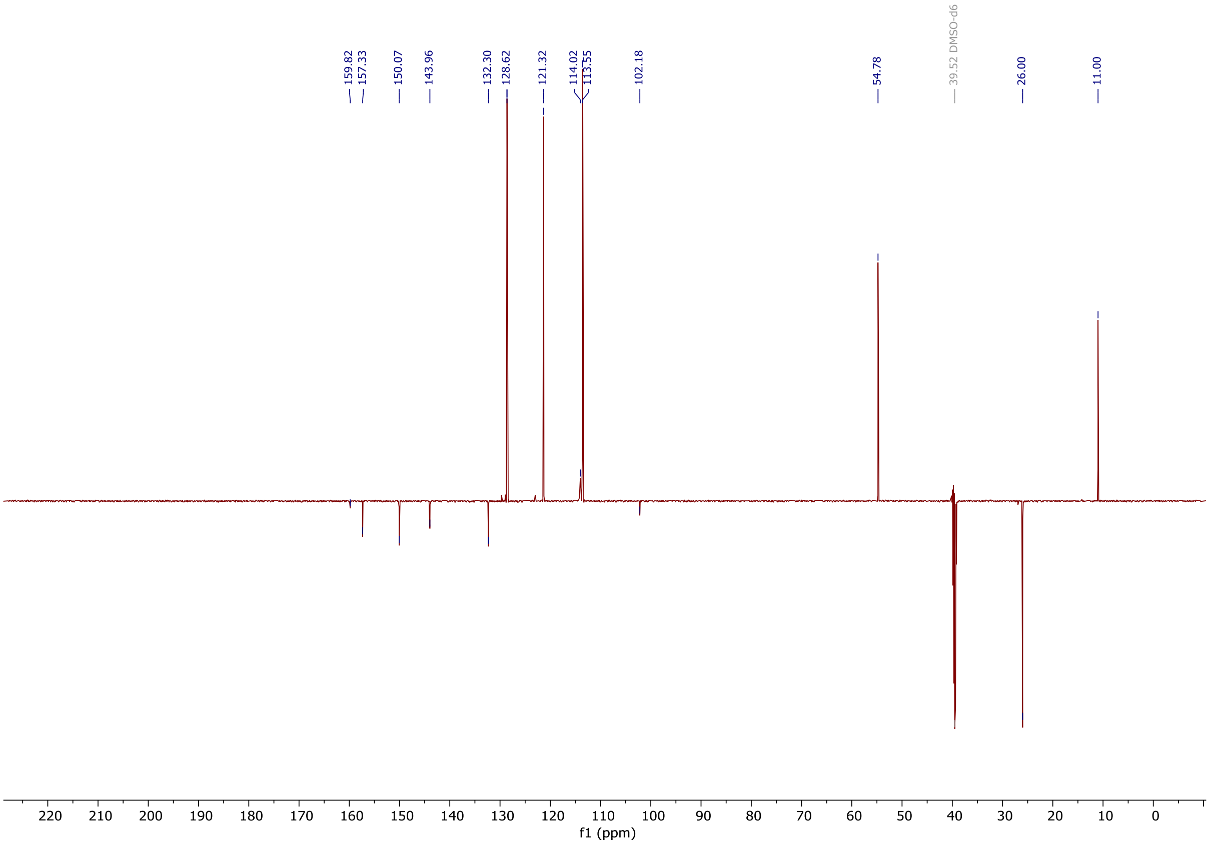

HMBC

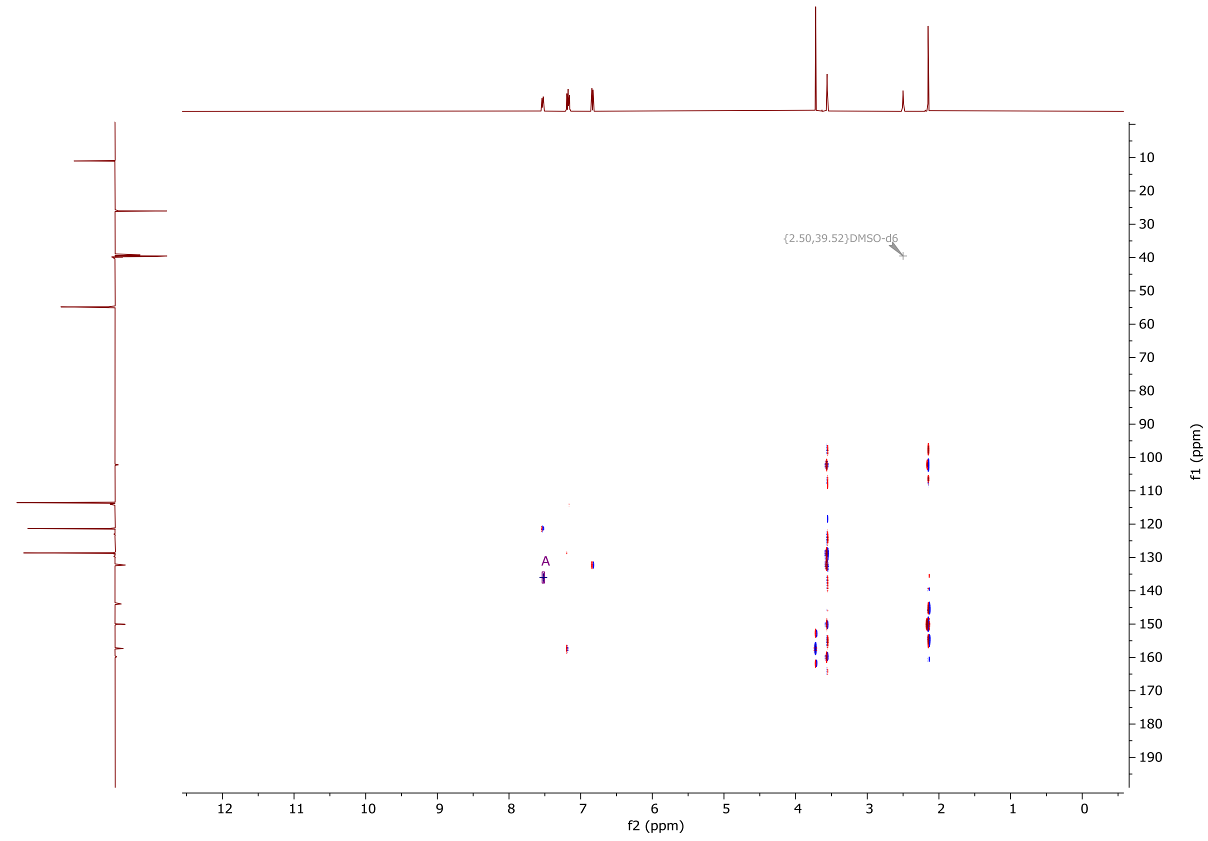

1-(1*H*-Benzo[*d*]imidazol-2-yl)-4-(4-methoxybenzyl)-3-(tetrahydro-2*H*-pyran-4-yl)-1*H*-pyrazol-5-ol (4)

^{1}H

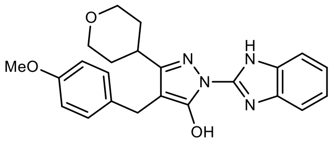

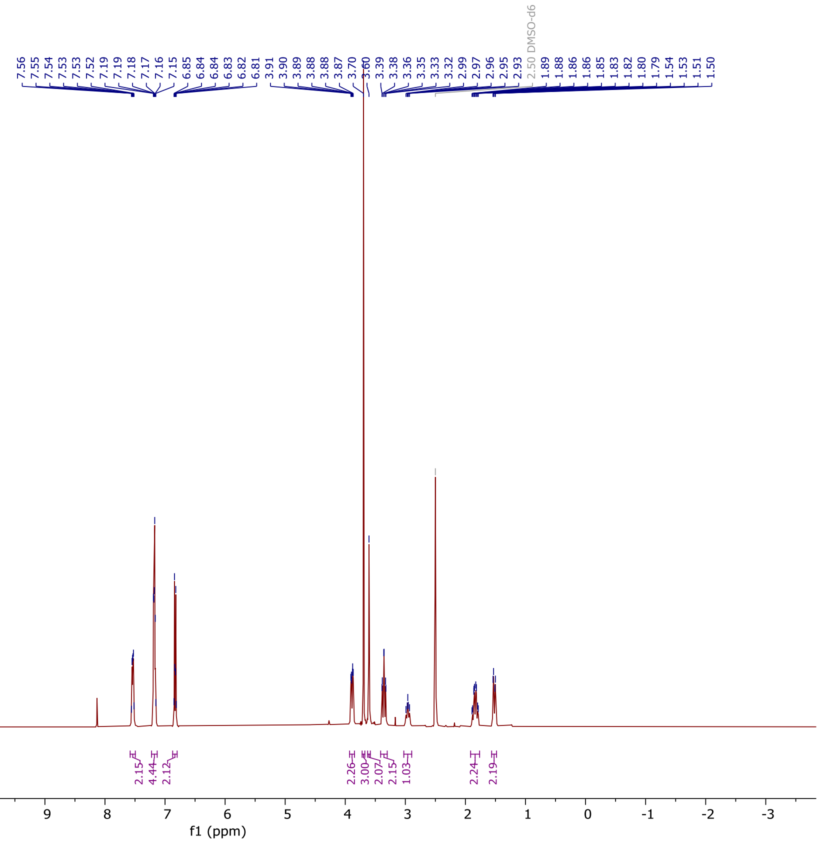

^{13}C

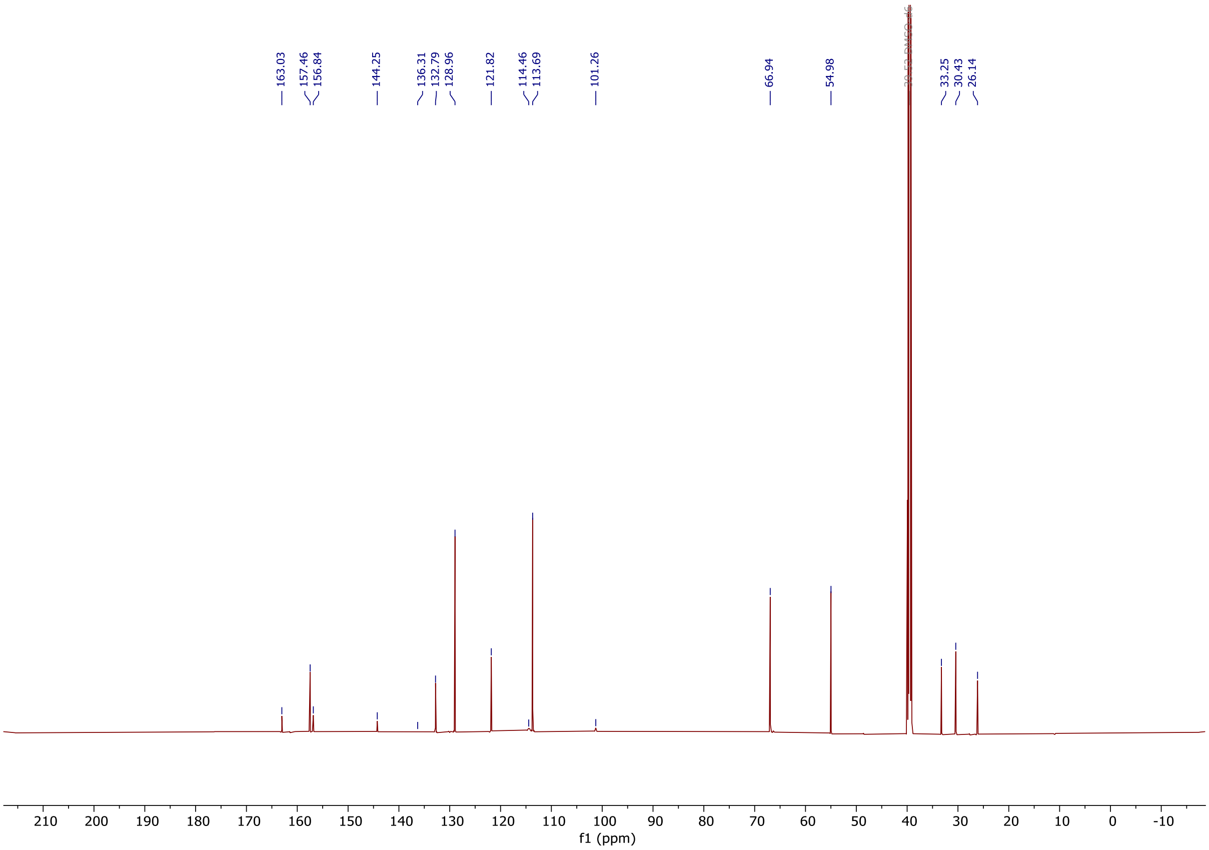
163.03
157.46
156.84
144.25
136.31
132.79
128.96
121.82
114.46
113.69
101.26
66.94
54.98
33.25
30.43
26.14
210
200
190
180
170
160
150
140
130
120
110
100
90
80
70
60
50
40
30
20
10
0
-10
f1 (ppm)

3-(5-Hydroxy-3-methyl-1-(5-nitro-1*H*-benzo[*d*]imidazol-2-yl)-1*H*-pyrazol-4-yl)isobenzofuran-1(3*H*)-one (5)

^{1}H

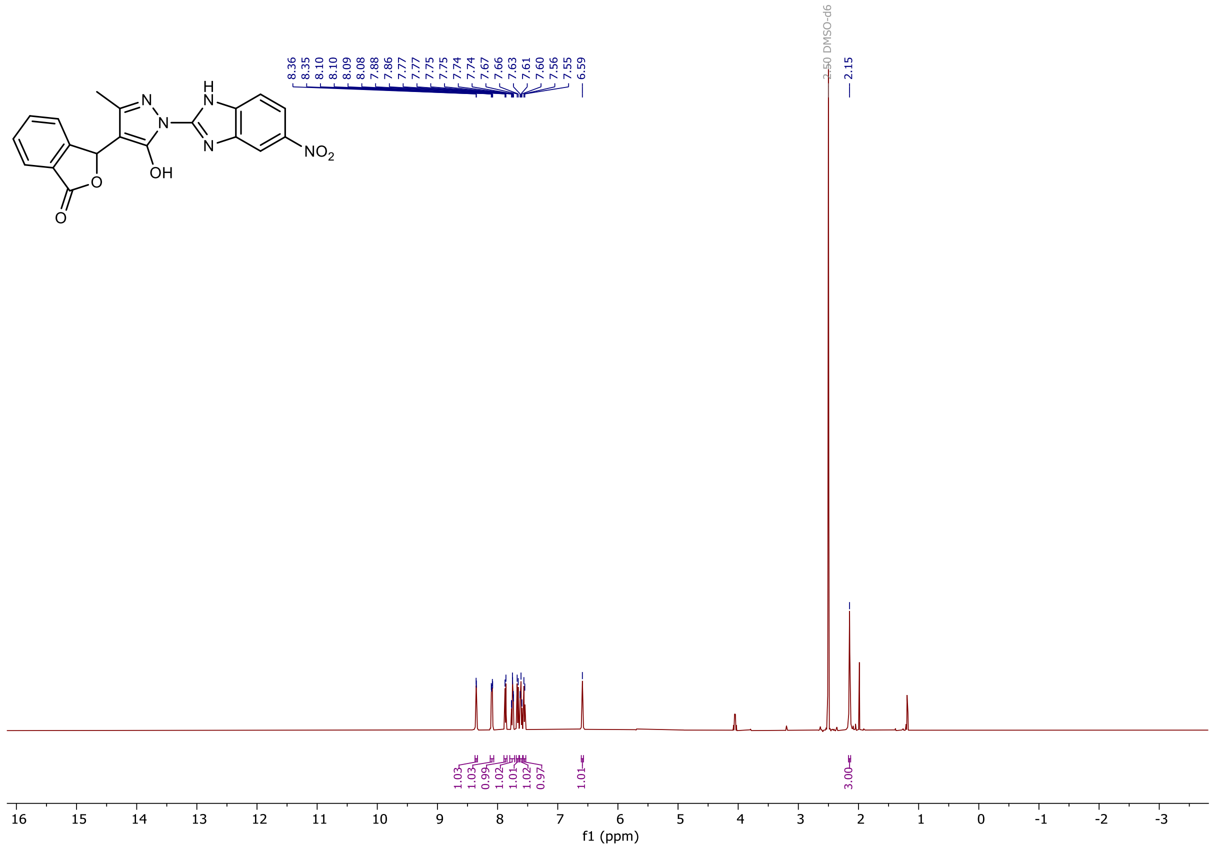

^{13}C DEPTq

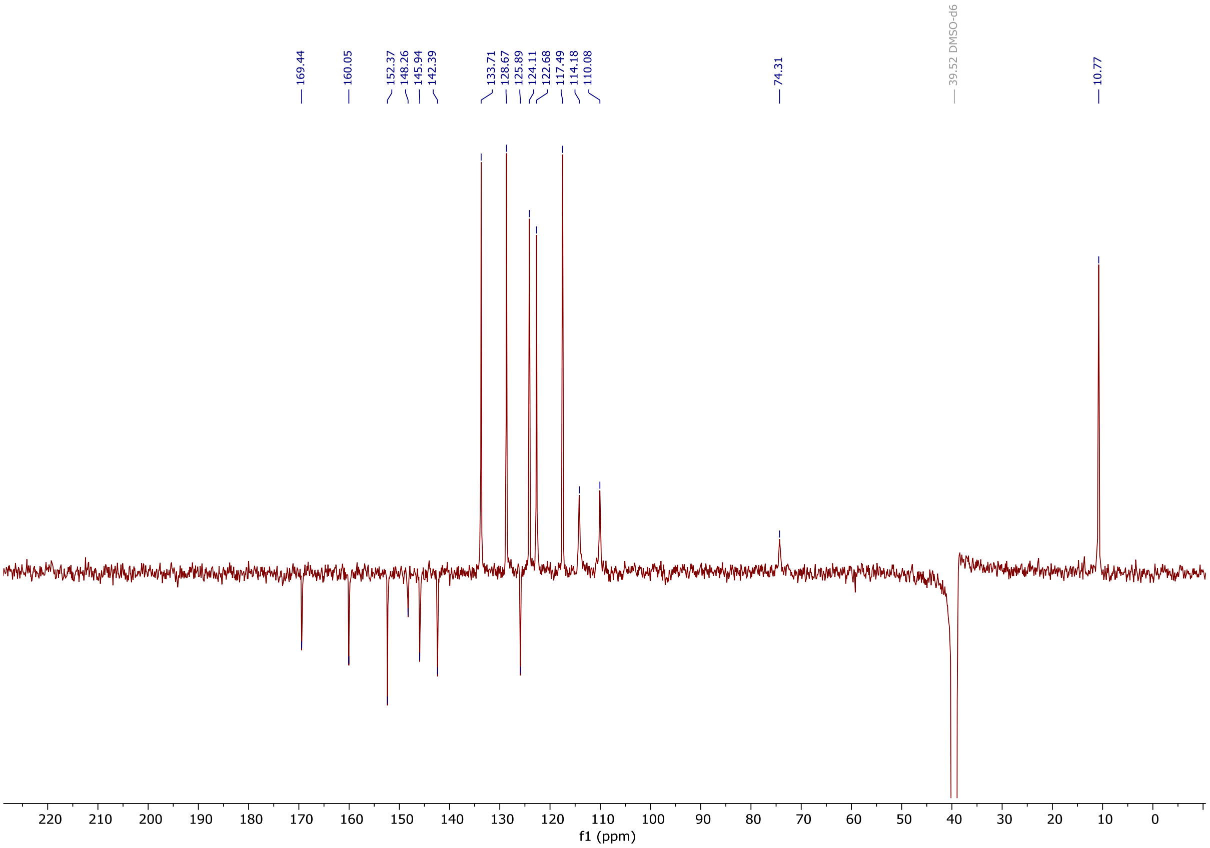

HMBC

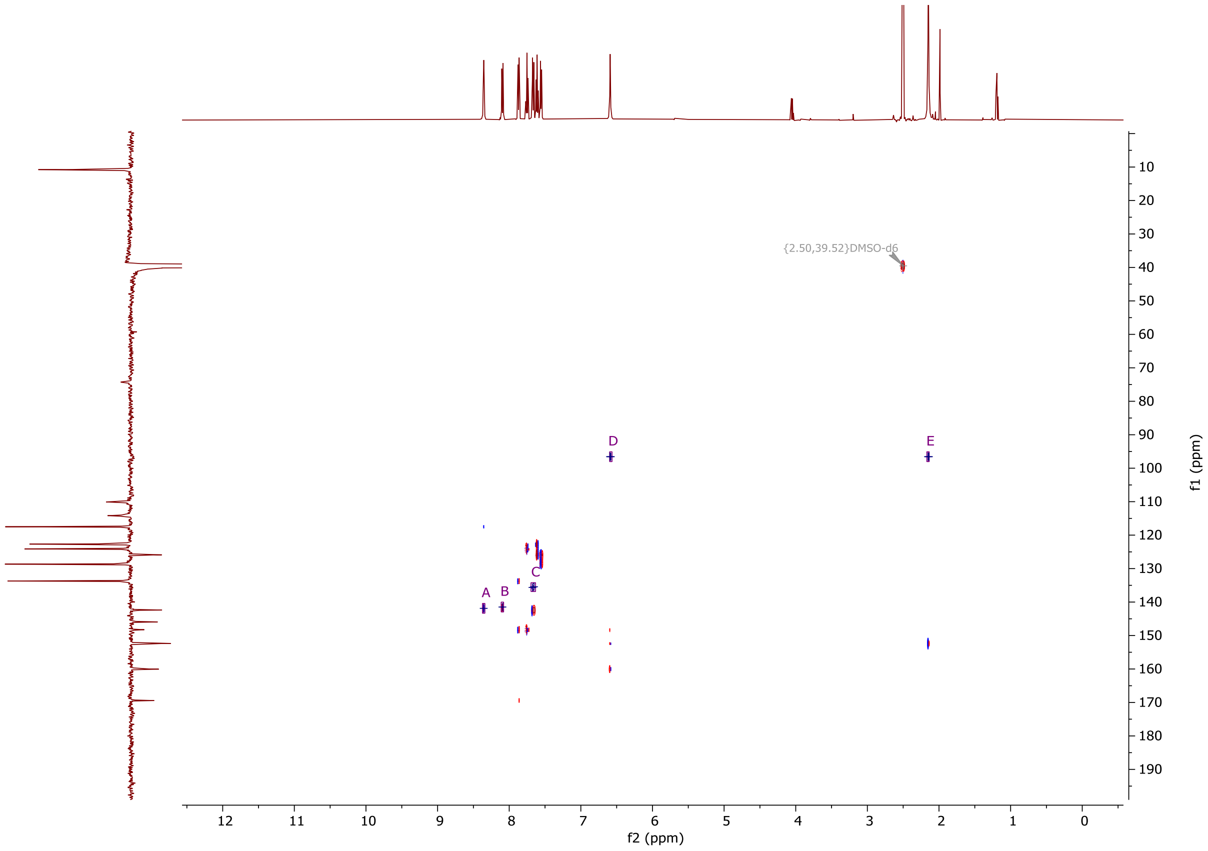

4-(4-Methoxybenzyl)-3-methyl-1-(5-nitro-1*H*-benzo[*d*]imidazol-2-yl)-1*H*-pyrazol-5-ol (6)

[1]H

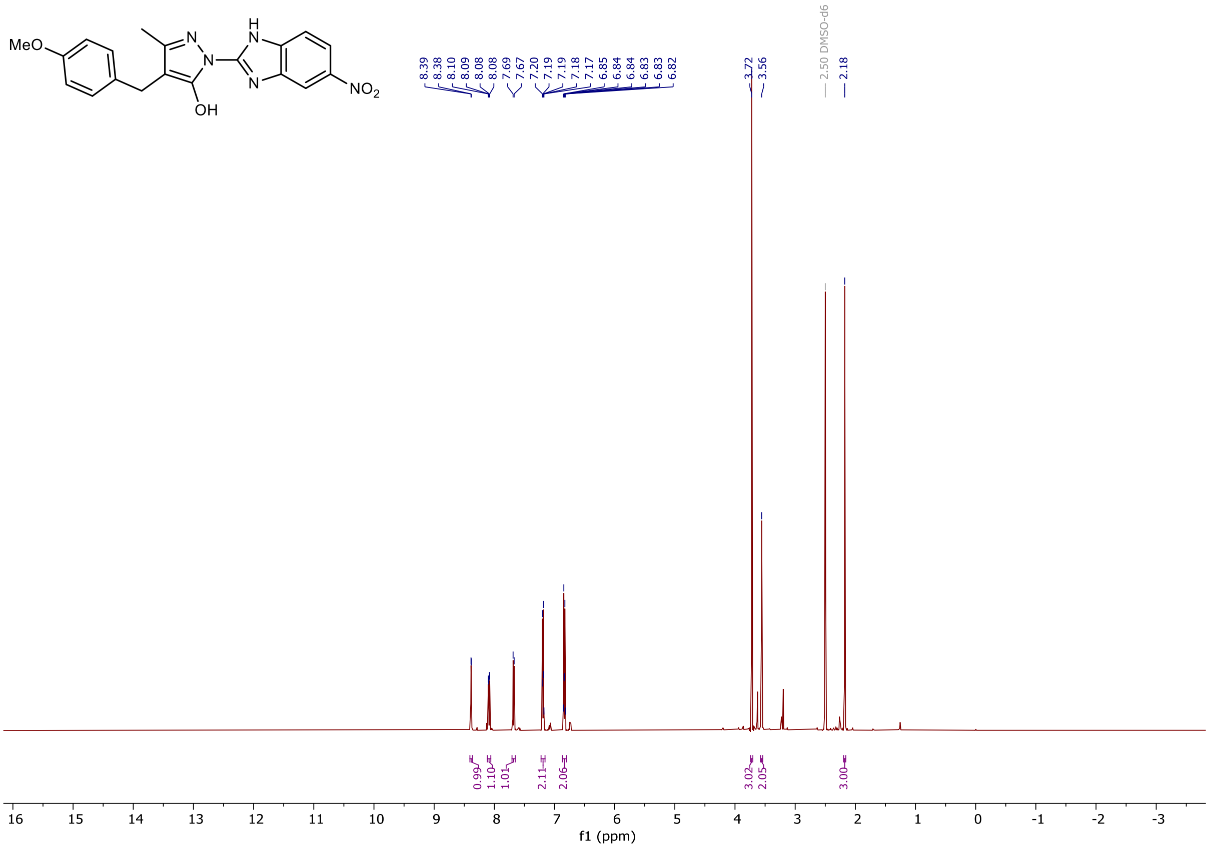

^{13}C DEPTq

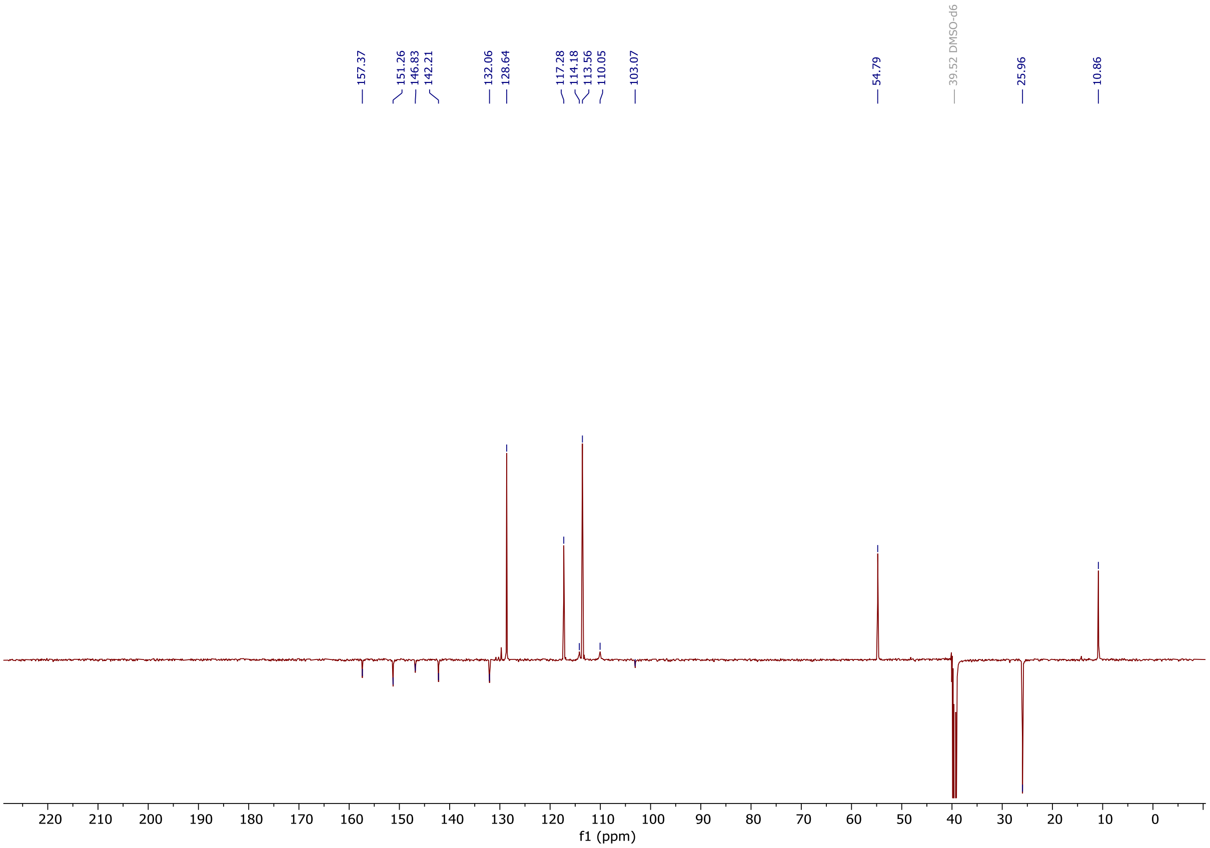

HMBC

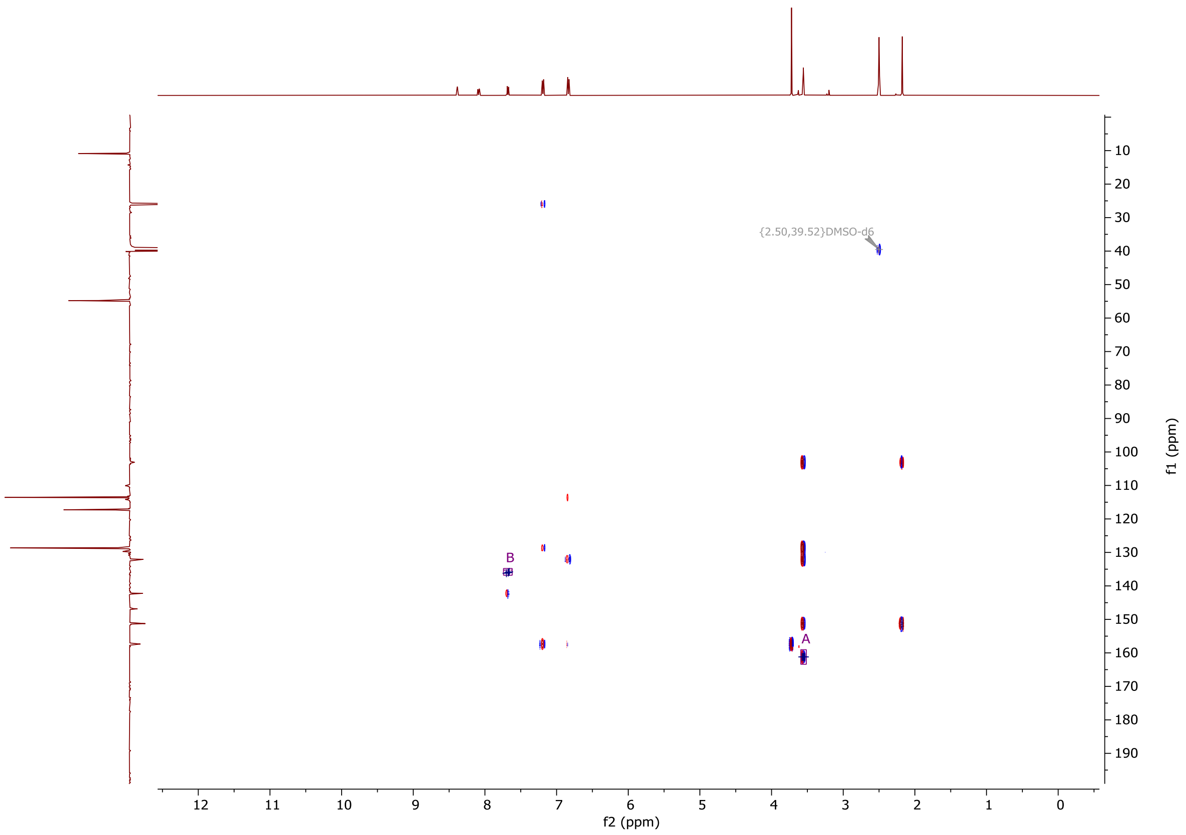

Supplementary references

1. J. W. Bondy-Denomy, The Diverse Impact of Bacteriophages on the Bacterial Host, PhD thesis, University of Toronto, 2014.
2. F. Su, Z. Sun, W. Su and X. Liang, NMR investigation and theoretical calculations on the tautomerism of benzimidazole compounds, *J. Mol. Struct.*, 2018, **1173**, 690-696.
